# Development of a near-infrared fluorescent probe for the selective detection of severe hypoxia†

**DOI:** 10.1039/d4cb00243a

**Published:** 2025-02-04

**Authors:** Takafumi Kasai, Kyohhei Fujita, Toru Komatsu, Tasuku Ueno, Ryosuke Kojima, Kenjiro Hanaoka, Yasuteru Urano

**Affiliations:** a Graduate School of Pharmaceutical Sciences, The University of Tokyo 7-3-1 Hongo, Bunkyo-ku Tokyo 113-0033 Japan uranokun@m.u-tokyo.ac.jp; b Graduate School of Medicine, The University of Tokyo 7-3-1 Hongo, Bunkyo-ku Tokyo 113-0033 Japan; c Graduate School of Pharmaceutical Sciences, Keio University 1-5-30 Shibakoen, Minato-ku Tokyo 105-8512 Japan

## Abstract

Severely hypoxic environments with oxygen concentrations around 1% are often found in serious diseases such as ischemia and cancer. However, existing near-infrared (NIR) fluorescent probes that can visualize hypoxia are also activated in mildly hypoxic environments (around 5% oxygen). Here, in order to selectively detect severe hypoxia, we used julolidine-based SiR (JSiR) as a NIR fluorophore and developed T-azoJSiR640 as a fluorescent probe. T-azoJSiR640 was able to detect severe hypoxia (around 1% oxygen concentration or less) in live cell imaging. Furthermore, the ischemic liver in a portal-vein-ligated mouse model was successfully visualized *in vivo*.

## Abstract 2

We have developed T-azoJSiR640 as a NIR fluorescence probe for severe hypoxia. In contrast to existing NIR probes for hypoxia, the probe was able to selectively visualize severe hypoxia in live cells and in ischemic liver of a mouse model *in vivo*.

## Graphical abstract

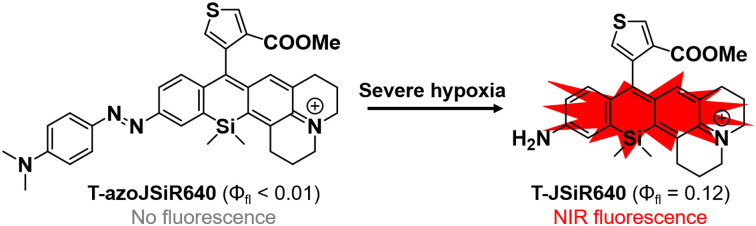

## Introduction

Hypoxia, generally considered as an oxygen concentration of <2%,^1^ is found in various diseases such as ischemia,^2,3^ chronic kidney disease^4,5^ and cancer.^6–9^ For example, hypoxia is one of the characteristics of tumor microenvironments and is known to promote DNA strand breaks while suppressing homologous recombination (HR)^10–12^ and mismatch repair (MMR).^10,13,14^ In addition, hypoxia-inducible factor 1α (HIF-1α) is activated under hypoxic conditions and regulates various downstream target genes,^8,10,15–17^ thereby promoting cancer cell proliferation,^17,18^ migration,^17–19^ invasion,^17–19^ and epithelial–mesenchymal transition (EMT).^17,20^ Indeed, oxygen concentrations as low as around 1% may occur in ischemia^21–23^ and cancer,^24,25^ and selective *in vivo* visualization of such severe hypoxia is especially important for monitoring and understanding these diseases. To analyze hypoxia in living cells and *in vivo*, various fluorescent probes have been developed.^26–30^ However, existing near-infrared (NIR) small molecular probes that can be effectively used *in vivo* are also activated at oxygen concentrations around 5%.^26,30–32^ Therefore, there is a need for the development of NIR fluorescent probes able to selectively detect severe hypoxia as *in vivo* analytical and diagnostic tools.

Our research group has focused on the reductive cleavage of azo groups under hypoxia and developed NIR probes based on fluorescence resonance energy transfer (FRET) between dicarbocyanines and black hole quencher (BHQ).^33^ Although these probes are selectively activated in severe hypoxia, they are not optimal for use in living cells and *in vivo* applications due to their large molecular size and susceptibility to photobleaching. As another strategy, we have recently developed fluorescent probes that detect hypoxia by incorporating an azo group into an O rhodamine or Si rhodamine (SiR) scaffold.^32,34^ For example, we have developed green-emitting MAR,^34^ red-emitting MASR^34^ and NIR-emitting azoSiR640 (2,6-diMe azoSiR640),^32^ and confirmed that they can detect hypoxia in cultured cells and mouse models. These fluorescent probes initially have almost no fluorescence due to fluorescence quenching by the azobenzene moiety incorporated in the fluorophore. Upon cleavage of the azo group by reductase-catalyzed reactions that are accelerated in hypoxia, the corresponding fluorescent rhodamines are produced, enabling selective detection of hypoxia. However, the oxygen concentration threshold of these rhodamine-based hypoxia probes varies depending on the fluorescent scaffold. For example, MASR and 2,6-diMe azoSiR640 (Fig. S1, ESI†) switched on at around 0.1% and 5% oxygen concentration, respectively.^32,34^ These characteristics may be due to differences in the rate of reverse oxidation of the azo moiety depending on the fluorescent scaffold (Fig. S2, ESI†). Thus, we considered that the development of NIR probes to selectively detect severe hypoxia of around 1% oxygen concentration might be achieved by using different fluorophores.

The mono-julolidine-fused analog of SiR (JSiR) is widely used as a NIR fluorophore for biological imaging.^35^ In this study, we therefore examined JSiR as the scaffold fluorophore of a NIR fluorescent probe for hypoxia. We also evaluated the responsiveness of the synthesized probe, T-azoJSiR640, to oxygen concentration and confirmed its utility to selectively detect severe hypoxia *in vivo*.

## Design and synthesis of compounds

**Fig. 1:**
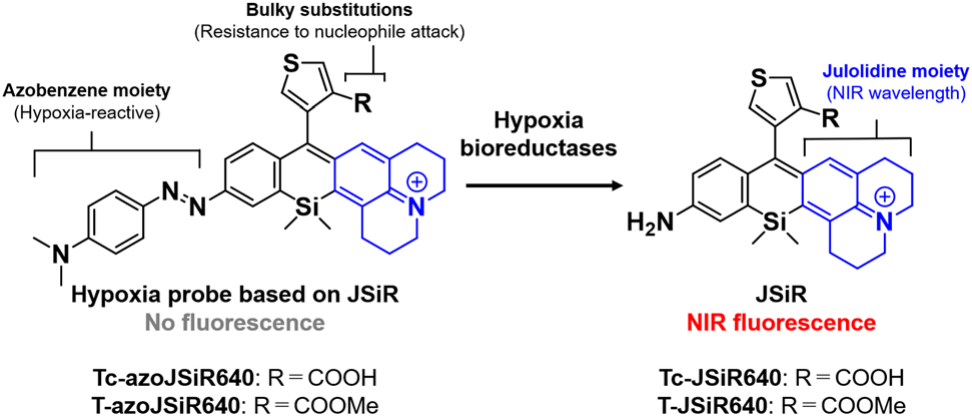
Molecular design of the NIR fluorescent probe for hypoxia based on JSiR.

**Scheme 1:**
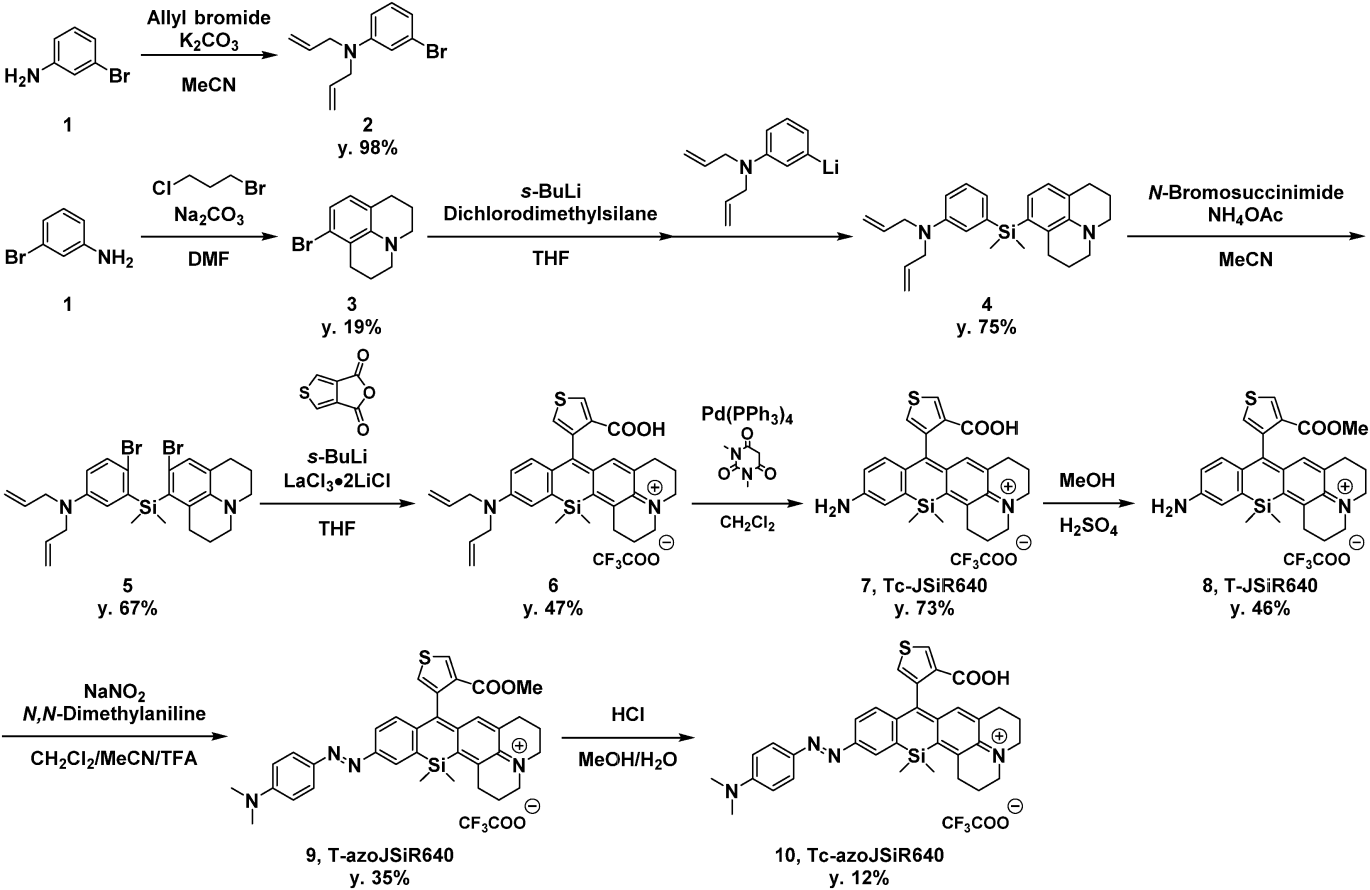
Synthetic scheme of T-azoJSiR640 and Tc-azoJSiR640.

Since the oxygen concentration threshold of the rhodamine-based hypoxia probe varies depending on the scaffold fluorophore,^32,34^ we focused here on JSiR as a widely used NIR fluorophore with different redox behavior from other SiR fluorophores. Building on our previously reported MASR^34^ and 2,6-diMe azoSiR640,^32^ we designed and developed a novel fluorescent probe for hypoxia by introducing an azobenzene moiety at a free amino group of JSiR (Fig. 1). Since bulky substitutions at the 2 or 6 positions of the benzene ring in SiR block nucleophilic attack at the 9 position of the xanthene ring,^32,36,37^ for example, by water and cysteine containing molecules, we firstly designed a probe possessing carboxylic acid at the 2 position of the benzene ring of JSiR. However, this proved synthetically difficult, requiring tedious synthetic and purification steps. So, we next designed a probe possessing a thiophene ring instead of the benzene ring. We synthesized this probe, Tc-azoJSiR640, as shown in Scheme 1, but it did not show activation in live cell imaging and proved ineffective (Fig. S3, ESI†). Since Tc-JSiR640 showed absorbance derived from the non-cyclized form at physiological and lysosomal pH, as well as in CH_2_Cl_2_, whose polarity is similar to that of internal cell membranes^38^ (Fig. S4 and S5, ESI†), the reason for this was considered to be poor cellular membrane permeability of Tc-azoJSiR640 due to the presence of the anionic form of the carboxylic acid moiety in its molecular structure.^39–41^ Thus, we decided to convert the carboxylic acid moiety to a methoxycarbonyl group and synthesized T-azoJSiR640 (Fig. 1 and Scheme 1). Compared to Tc-azoJSiR640, the membrane permeability of T-azoJSiR640 was apparently improved, as described later. Therefore, we focused on T-azoJSiR640 and evaluated its optical properties and susceptibility to hypoxia.

## Optical properties and stability of the developed probes

**Fig. 2:**
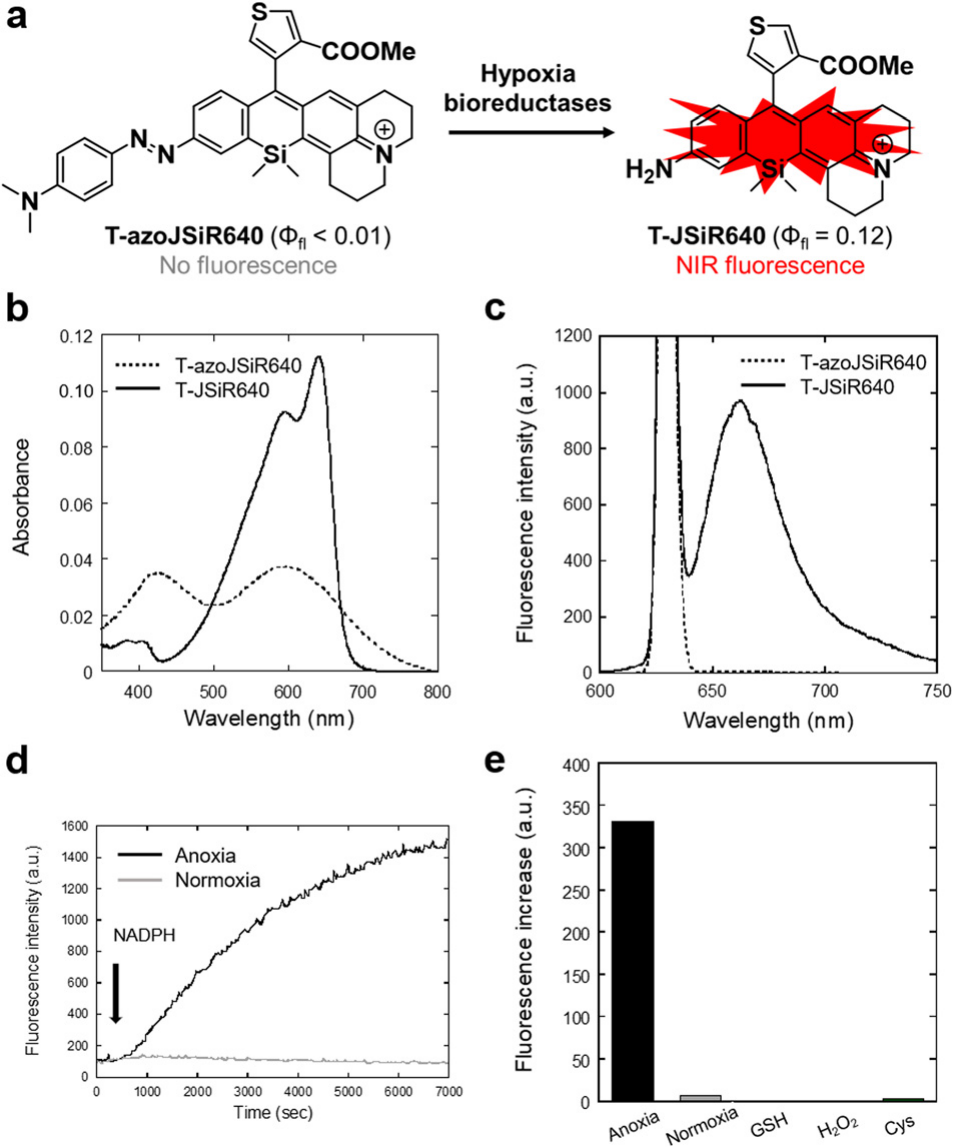
Developed NIR fluorescent probe for hypoxia, T-azoJSiR640. (a) NIR fluorescence activation of T-azoJSiR640. T-azoJSiR640 is non fluorescent, but is converted to fluorescent T-JSiR640 by bioreductases under hypoxia. (b) Absorbance spectra of T-azoJSiR640 and T-JSiR640. [T-azoJSiR640] = 1.0 μM, [T-JSiR640] =1.0 μM. Both spectra were measured in 100 mM sodium phosphate buffer at pH 7.4 including 0.1% MeOH or 0.1% DMSO, respectively. (c) Fluorescence spectra of T-azoJSiR640 and T-JSiR640. [T-azoJSiR640] = 0.3 μM, [T-JSiR640] = 0.3 μM. Both spectra were measured in 100 mM sodium phosphate buffer at pH 7.4 including 0.1% MeOH or 0.1% DMSO, respectively. The excitation wavelength was 630 nm. (d) Time-dependent fluorescence changes of T-azoJSiR640 in the presence of rat liver microsomes under anoxia and normoxia at 37 °C. Assays were performed in 100 mM sodium phosphate buffer at pH 7.4 containing 0.5% MeOH as a cosolvent. The anoxia environment was prepared by bubbling Ar gas for 30 min. NADPH was added at 4 min. The excitation and emission wavelengths were 630 nm and 650 nm. [T-azoJSiR640] = 3.0 μM, [rat liver microsomes] = 76 μg mL^−1^, [NADPH] = 128 μM. (e) Fluorescence increases of T-azoJSiR640 after incubation for 1 hr under various conditions. Each measurement was performed at 37 °C in 100 mM sodium phosphate buffer at pH 7.4 containing 0.5% MeOH as a cosolvent. The anoxic environment was prepared by bubbling Ar gas for 30 min and waiting for 30 min. The anoxia and normoxia assays were performed in the presence of rat liver microsomes and NADPH. The excitation and emission wavelengths were 630 nm and 662 nm. [T-azoJSiR640] = 2.6 μM, [rat liver microsomes] = 71 μg mL^−1^, [NADPH] = 50 μM. [GSH] = 5 mM, [H_2_O_2_] = 50 μM, [Cys] = 50 μM.

We next evaluated the optical properties of the synthesized T-azoJSiR640 and T-JSiR640 (Table 1). T-JSiR640 was expected to be produced from T-azoJSiR640 under reductive conditions (hypoxia) (Fig. 2(a)). T-azoJSiR640 showed a broad absorbance spectrum with almost no fluorescence in sodium phosphate buffer (100 mM; pH 7.4) (Fig. 2(b) and (c)). The observed complete quenching of fluorescence was considered to be due to ultrafast *cis*/*trans* conformational change of the azo bond of T-azoJSiR640 in the excited state, as in the case of our previously reported probes.^32–34^ On the other hand, T-JSiR640 showed marked fluorescence with a peak at 662 nm in the NIR range (Fig. 2(c)), and its fluorescence quantum yield was 0.12 (Table 1). We also confirmed that T-JSiR640 showed essentially the same fluorescence spectra over the pH range from 5.0 to 9.0, indicating that the fluorescence signal of T-JSiR640 is not affected by pH (Fig. S6, ESI†). These results suggested that T-azoJSiR640 is able to generate NIR fluorescence upon cleavage of the azo group under hypoxia.

**Table 1:** Photophysical properties of T-azoJSiR640 and T-JSiR640 in sodium phosphate buffer at pH 7.4 including 0.1% MeOH or 0.1% DMSO, respectively

	*λ*_abs_ [nm]	*λ*_fl_ [nm]	*Φ*_fl_
T-azoJSiR640	595	n.d.	<0.01
T-JSiR640	641	662	0.12

## Rat liver microsome assay

To determine whether T-azoJSiR640 could detect anoxia, we first performed an *in vitro* assay using rat liver microsomes, which contain various reductases such as NADPH-cytochrome P450 reductase. The probe T-azoJSiR640 showed a fluorescence increase only in the presence of rat liver microsomes and NADPH under anoxia, indicating that the probe could detect anoxia under reducing conditions catalyzed by microsomal reductases (Fig. 2(d)). Furthermore, the probe T-azoJSiR640 showed a negligible fluorescence increase in the presence of other biospecies such as GSH, H_2_O_2_ and Cys (Fig. 2(e)).

## Live cell fluorescence imaging of hypoxia

**Fig. 3:**
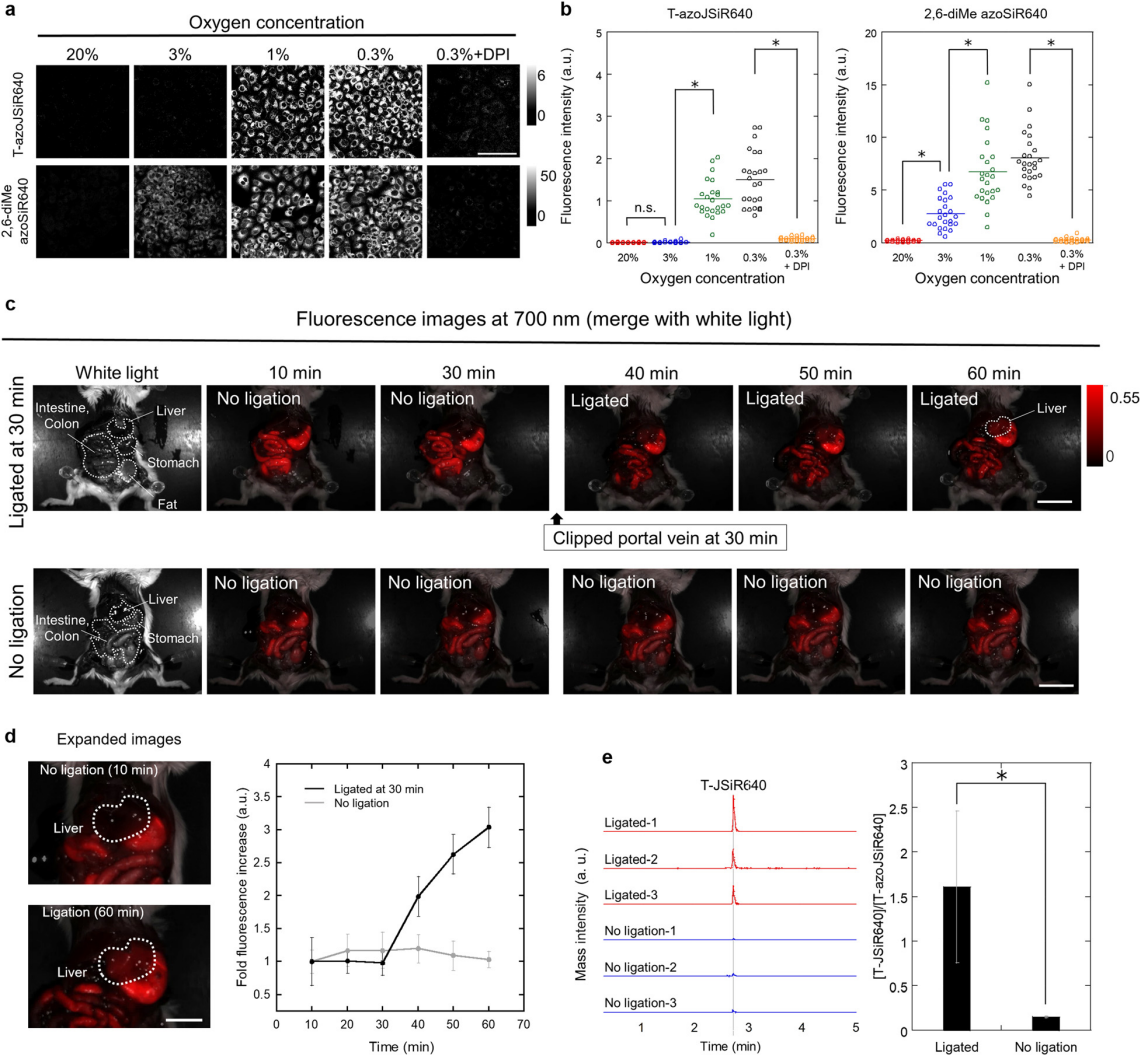
Live cell and *in vivo* fluorescence imaging of hypoxia. (a) Fluorescence images of A549 cells incubated with T-azoJSiR640 and 2,6-diMe azoSiR640 under various oxygen concentrations (20%, 3%, 1% and 0.3%). The inhibitor experiment was performed by the addition of the probe and DPI under 0.3% oxygen concentration. All fluorescence images were captured 6 h after administration of the probe. Fluorescence imaging was performed in phenol red (–) DMEM containing 10% FBS, 1% PS and 1% DMSO as a cosolvent. The excitation and emission wavelengths were 630 nm/650–750 nm. [T-azoJSiR640] = 1 μM. [2,6-diMe azoSiR640] = 1 μM. [DPI] = 30 μM. Scale bars = 100 μm. (b) The dot-plot diagram of the fluorescence intensities of a single-cell at each oxygen concentration (*n* = 24). The line in the diagram represents the average. The significance of the differences was determined by one-way ANOVA with Tukey's HSD (*P < 0.01). n.s. = not significant. (c) *In vivo* fluorescence imaging of liver ischemia of mouse models. 200 μM T-azoJSiR640 in PBS (150 μL) containing 4% ethanol (v/v) as a cosolvent was administered by intravenous injection. The portal vein was ligated with thread at 30 min after probe injection to induce liver ischemia. The liver showed a NIR fluorescence increase only after induction of ischemia. The excitation and emission wavelengths were 570–630 nm/650-nm. Scale bars = 2 cm. (d) *In vivo* fluorescence imaging of liver ischemia of mouse models (left). Scale bars = 1 cm. Fold fluorescence increase of the liver at 700 nm (*n* = 3) (right). Fold fluorescence increase represents the increase at 10 min after probe administration. Ischemic liver showed a specific fluorescence increase. Error bar represents S.D. (e) LC-MS/MS chromatograms of T-JSiR640 in ischemic liver and liver without portal vein ligation from the *in vivo* imaging experiment (*n* = 3). Production of T-JSiR640 was selectively detected in ischemic liver. *P < 0.05 by student's *t*-test. Error bar represents S.D.

To evaluate whether T-azoJSiR640 could visualize hypoxia in living cells, we used it to perform live-cell fluorescence imaging of A549 lung adenocarcinoma cells under various oxygen concentrations. T-azoJSiR640 showed almost no fluorescence under normoxia (Fig. 3(a), (b) and Fig. S7, ESI†), while it showed a significant increase of NIR fluorescence at oxygen concentrations of around 1%. These results suggested that T-azoJSiR640 was able to selectively detect severe hypoxia. The activation of T-azoJSiR640 in living cells also indicated that this probe showed greater membrane permeability than Tc-azoJSiR640. In contrast with the above results, our previously reported 2,6-diMe azoSiR640 was also activated at around 5% oxygen concentration (Fig. 3(a), (b) and Fig. S8, ESI†). To confirm that the observed fluorescence signals were due to reduction by flavoproteins, we measured fluorescence images in the presence of a NADPH oxidase inhibitor, diphenyleneiodonium chloride (DPI).^42^ The fluorescence increase was dramatically suppressed in the presence of DPI, suggesting that T-azoJSiR640 is reduced by flavoproteins such as NADPH-cytochrome P450 reductase.

We assumed that this selective activation of T-azoJSiR640 around 1% oxygen concentration is due to the high rate of reverse oxidation of the azo anion radical produced in the first reduction step (Fig. S2, ESI†). This would explain why the fluorescence signal of T-azoJSiR640 is suppressed around 20–3% oxygen concentration, but is activated in extremely low oxygen concentration (around 1%), thereby allowing the selective detection of severe hypoxia.

## *In vivo* fluorescence imaging of liver ischemia in mouse models

Finally, we performed fluorescence imaging of liver ischemia of a mouse to determine whether T-azoJSiR640 could detect hypoxia *in vivo*. The probe T-azoJSiR640 was administered to mice by intravenous injection, and after 30 min, the portal vein was ligated to induce ischemia. Before ligation of the portal vein, almost no fluorescence increase was observed in the liver, while a rapid and significant fluorescence increase was observed over the entire liver after the induction of ischemia (Fig. 3(c) and (d)). To confirm that T-JSiR640 was produced, we examined the production of T-JSiR640 by LC-MS/MS analysis (Fig. 3(e) and Fig. S9, ESI†). Indeed, T-JSiR640 was selectively generated in liver ischemia, while it was not detected in the liver of mice without portal vein ligation (Fig. 3(c)), indicating that T-azoJSiR640 was selectively activated in ischemic tissues. These results demonstrated that T-azoJSiR640 can visualize hypoxia *in vivo*.

## Conclusions

We have designed, synthesized, and evaluated T-azoJSiR640 as a NIR fluorescent probe for severe hypoxia. In contrast to existing NIR probes for hypoxia, T-azoJSiR640 was able to selectively visualize severe hypoxia (around 1% oxygen concentration or less) in live cells and in the ischemic liver of a mouse model *in vivo*. Our results demonstrated that the developed NIR fluorescent probe T-azoJSiR640 has the potential to be a useful tool for analysing, monitoring and understanding various hypoxia-related diseases. For example, in many types of cancer, oxygen levels have been reported to decrease to as low as 1%. Under such conditions, various processes such as cancer cell proliferation, migration, invasion, and EMT are highly induced through the activation of HIF-1α. The selective imaging of severe hypoxia using T-azoJSiR640 is expected to facilitate a more detailed analysis of these biological processes and disease mechanisms.

## Experimental

### General procedures and materials

Reagents and solvents were of the best grade available, purchased from Tokyo Chemical Industries, FUJIFILM Wako Pure Chemical Corporation, Sigma-Aldrich, Kanto Chemical Co., Watanabe Chemical Industries, and were used without further purification. Reactions were monitored by means of TLC, ESI mass spectrometry and HPLC. All compounds were purified by silica gel chromatography or preparative HPLC.

### Instrument

NMR spectra were recorded on a JNM-ECZ400S instrument at 400 MHz for ^1^H NMR and at 101 MHz for ^13^C NMR. Mass spectra (MS) were measured with a JEOL JMS-T100LC AccuToF (ESI^+^). HPLC analyses were performed on an Acquity UPLC H-Class system (Waters) equipped with an Acquity UPLC BEH C18 1.7 μm (2.1  ×  50 mm) column (Waters) and an MS detector QDa (Waters). Preparative HPLC was performed on an Inertsil ODS-3 5 μm (10.0 × 250 mm) column (GL Sciences, Tokyo, Japan) using an HPLC system composed of a pump (PU-2086, JASCO) and a detector (MD-2015 or FP-2025, JASCO), or on Sfär C18 D – Duo 100 Å 30 μm 12 g or 30 g (Biotage) using Isolera™ One (Biotage). Eluent A (H_2_O containing 0.1% TFA), eluent B (80% MeCN and 20% H_2_O containing 0.1% TFA), eluent C (H_2_O containing 10 mM ammonium formate), eluent D (MeCN containing 0.1% TFA) and eluent E (MeCN) were used for HPLC purification. Column chromatography using silica gel was performed on an MPLC system (Yamazen Smart Flash EPCLC AI-580S) and a silica gel 40 μm column (Yamazen).

### HPLC analysis for reaction monitoring

HPLC analyses were performed on an Acquity UPLC H-Class system (Waters) equipped with an Acquity UPLC BEH C18 1.7 μm (2.1  ×  50 mm) column (Waters) and an MS detector QDa (Waters). Eluent C and eluent E, or eluent F (H_2_O containing 0.1% formic acid) and eluent G (MeCN containing 0.1% formic acid) were used.

### Optical properties and fluorescence quantum efficiency

Fluorescence spectra were obtained with a Hitachi F-7000 or F-7100. The slit width was 5 nm for both excitation and emission. The photomultiplier voltage was 700 V. UV-visible absorption spectra were obtained with a Shimadzu UV-1850 or UV-2450. Fluorescence quantum efficiency was measured with a Quantaurus-QY (Hamamatsu Photonics) in 100 mM sodium phosphate buffer at pH 7.4.

### Probe stock solution

T-azoJSiR640 was weighed, dissolved in methanol and aliquoted to 1.5 mL Microtube Black for Shading (WATSON® BIO LAB). The solvent was evaporated and the tubes were stored at −25 °C. The samples were reinstated in an appropriate solvent before use. The probe solution was prepared individually for each experiment and not reused. T-JSiR640 and Tc-JSiR640 were solved in DMSO and stored in 1.5 mL Safe-Lock Tubes at −25 °C. The concentrations of the stock solutions were calculated from the *ε* values. The concentration of Tc-JSiR640 was adjusted using the same *ε* values as T-JSiR640.

### Preparation of rat liver microsomes

All animal experiments were performed according to institutional guidelines. Rats (Wistar, boar, 6–7 weeks old at the beginning of the experiment) were purchased from CLEA Japan. They were treated with 60 mg/5 mL kg^−1^ sodium phenobarbital intraperitoneally once daily for 3 days, fasted overnight, and sacrificed by exsanguination from the abdominal aorta. The livers containing 0.15 M KCl at pH 7.4 were homogenized in 3 equal volumes of the same buffer and centrifuged (8500 rpm, 20 min, 4 °C) twice. Then the supernatant was collected and centrifuged (34 000 rpm, 80 min, 4 °C) to collect rat liver microsomal fractions. Rat liver microsomes contained 71.2 mg protein per mL and 0.479 nmol P450 per mg protein. The microsome fraction was diluted in 100 mM sodium phosphate buffer at pH 7.4 for assay.

### Rat liver microsome assay

The anoxia condition was prepared in a sealed cuvette. The assay solution (2 mL of 100 mM sodium phosphate buffer at pH 7.4) containing 3 μM T-azoJSiR640, rat liver microsomes (76 μg per mL) and 0.5% MeOH was bubbled with Ar gas for 30 min. As a reductase cofactor, 128 μM NADPH which had been previously bubbled with Ar gas for 30 min at 0 °C was added at 240 s and fluorescence intensity was measured over 7000 s at 37 °C. The excitation and emission wavelengths were 630 nm and 650 nm.

### Fluorescence increases under other conditions (GSH, H_2_O_2_ and Cys)

The anoxia condition was prepared in a sealed cuvette. The assay solution (3 mL of 100 mM sodium phosphate buffer at pH 7.4) containing 2.6 μM T-azoJSiR640, rat liver microsomes (71 μg mL^−1^), NADPH (50 μM), and 0.5% MeOH was bubbled with Ar gas for 30 min and left for 30 min. The normoxia condition was performed in the presence of 2.6 μM T-azoJSiR640, rat liver microsomes (71 μg mL^−1^), NADPH (50 μM), and 0.5% MeOH. For GSH, H_2_O_2_ and Cys conditions, each assay was performed in 100 mM sodium phosphate buffer at pH 7.4 containing 2.6 μM T-azoJSiR640 and MeOH 0.5% including GSH (5 mM), H_2_O_2_ (50 μM) or Cys (50 μM). The fluorescence intensities of T-azoJSiR640 were measured at 0 min and 1 h. The excitation and emission wavelengths were 630 nm and 662 nm (Fig. 2(e)).

### Cell lines and culture conditions

A549 lung adenocarcinoma cells were purchased from the RIKEN BioResource Center Cell Bank. A549 cells were cultured in DMEM containing 10% FBS and 1% PS at 37 °C under 5% CO_2_ air.

### Live cell fluorescence imaging of hypoxia

A549 cells (3.0 × 10^4^ cells, passage >3) were seeded on 8-chamber plates (Nippon Genetic Co., Ltd) and cultured for 1 day before assay. On the next day, DMEM was removed and 200 μL phenyl red (−) DMEM supplemented with 10% FBS and 1% PS containing 1 μM probe and 1% DMSO as a co-solvent was added. The cells were incubated for 6 h under various oxygen concentrations (0.3, 1, 3, 20%) and 5% CO_2_ at 37 °C (Fig. 3(a) and Fig. S7, S8, ESI†). The oxygen concentrations were controlled with a MCO-5MUV (Sanyo) multi gas incubator by N_2_ substitution. Fluorescence confocal microscopy images were acquired using a Leica application suite advanced fluorescence (LAS-AF) instrument equipped with a TCS SP5 and 40× or 10× objective lens. Gain and pinhole values were set at 150% and 95.0 μm (Fig. 3(a) and Fig. S7, S8, ESI†) or 68.0 μm (Fig. S3, ESI†). The frame-accumulation was 2, the line-average was 16 and the resolution was 8 bits. The light source was a white light laser. The excitation and emission wavelengths were 630 nm/650–750 nm. The inhibitor experiment was performed by adding 30 μM DPI. Four images from different wells were captured under each measurement condition, and six cells per image were randomly selected. The fluorescence intensity of these cells was quantified by drawing regions of interest (ROI) using Image-J software (Fig. S7 and S8, ESI†).

### *In vivo* fluorescence imaging of liver ischemia in mouse models

All animal procedures were performed in accordance with the guidelines for Care and Use of Laboratory Animals of the University of Tokyo and approved by the Animal Ethics Committee of the University of Tokyo. Female Jcl: ICR mice (7 weeks) were used. T-azoJSiR640 (200 μM) in PBS (150 μL) containing 4% ethanol was administered by intravenous injection. After probe administration, the mice were anesthetized with a combination of domitor, butorphanol and midazolam. Fluorescence images were captured at 10 min, 20 min and 30 min after administration of the probe, and then the portal vein was ligated with suture thread at 30 min. Further fluorescence images were captured at 40 min, 50 min and 60 min. All fluorescence images were captured with the CRi Maestro imaging system (CRi Inc., Woburn, MA). Exposure time was 70 ms. The stage was set at 1B. The emission filter was longpass filter/VIS 650 nm (Asahi Spectra Co., Ltd) and the excitation filter was bandpass filter/600 nm (Asahi Spectra Co., Ltd). Fluorescence at 700 nm was extracted, and fluorescence intensities were quantified by drawing the region of interest (ROI) with Maestro software.

### LC-MS/MS analysis of the resected liver

LC-MS/MS analysis was performed using the LC system Acquity UPLC H-Class (Waters) equipped with an Acquity UPLC BEH C18 1.7 μm (2.1 × 50 mm) column (Waters) and an MS/MS detector (Xevo TQD, Waters). Eluent F (H_2_O containing 0.1% formic acid) and eluent H (80% MeCN and 20% H_2_O containing 0.1% formic acid) were used with the following binary linear gradient conditions: F/H = 95/5 isocratic for 0.3 min, then linearly shifted to 5/95 over 2.7 min. Flow rate was 0.8 mL min^−1^. MS detection was in the positive ion mode with multiple reaction monitoring (MRM) settings. The frozen liver sections (50–100 mg) were extracted with 10 volumes of 80% MeCN and 20% H_2_O containing fluorescein (1 μM) as an internal standard. The extract was centrifuged (14 000 rpm, 10 min, 4 °C), and the supernatant was collected and stored at 0 °C before analysis. 5 mL of sample was used for the analysis, and the expected concentrations were calculated from the calibration curve prepared by independently injecting T-JSiR640 and T-azoJSiR640. For MRM analysis, *m*/*z* = 59.1 > 473.2, 89.0 > 473.2 (cone voltage = 50 V, collision energy = 90 V) was monitored for T-JSiR640, and *m/z* = 89.0 > 605.2, 120.1 > 605.2, 456.2 > 605.2 (cone voltage = 30 V, collision energy = 90 V) was monitored for T-azoJSiR640.

## Abbreviations

AcOEt: Ethyl acetate
AcOH: Acetic acid
Ar: Argon
CH_2_Cl_2_: Dichloromethane
Cys: Cysteine
DMEM: Dulbecco's modified Eagle's medium
DMF: *N,N*-Dimethylformamide
DMSO: Dimethyl sulfoxide
DPI: Diphenyleneiodonium chloride
ESI: Electrospray ionization
*ε*
: Molar extinction coefficient
FBS: Fetal bovine serum
*Φ*_fl_
: Fluorescence quantum efficiency
GSH: Glutathione
HCl: Hydrogen chloride
H_2_O: Water
H_2_O_2_: Hydrogen peroxide
HPLC: High-performance liquid chromatography
K_2_CO_3_: Potassium carbonate
LaCl_3_·2LiCl: Lanthanum(iii) chloride bis(lithium chloride) complex
LC/MS: Liquid chromatography mass spectrometry
MeCN: Acetonitrile
MeOH: Methanol
Na_2_SO_4_: Sodium sulfate
Na_2_S_2_O_3_: Sodium thiosulfate
NADPH: Nicotinamide adenine dinucleotide phosphate
NaHCO_3_: Sodium hydrogen carbonate
NaNO_2_: Sodium nitrite
NH_4_OAc: Ammonium acetate
NMR: Nuclear magnetic resonance
N_2_: Nitrogen
PBS: Phosphate-buffered saline
Pd(PPh_3_)_4_: Tetrakis(triphenylphosphine)palladium (0)
PS: Penicillin–streptomycin
*s*-BuLi: *sec*-Butyllithium
TLC: Thin layer chromatography
TFA: Trifluoroacetic acid
THF: Tetrahydrofuran.

## Author contributions

Takafumi K., K. F., K. H. and Y. U. wrote and reviewed the manuscript. Takafumi K. synthesized the compounds. Takafumi K. performed rat liver microsome assays. Takafumi K. and K. F. performed live cell fluorescence imaging and the date analyses. Takafumi. K., K. F. and Toru K. performed *in vivo* fluorescence imaging and data analyses. K. F., Toru K., T. U., R. K., K. H. and Y. U. discussed the results of experimental data and supported the experiments. Y. U. supervised the entire project.

## Data availability

The data supporting this article have been included as part of the ESI.† Raw data were generated at The University of Tokyo. Derived data supporting the findings of this study are available from the corresponding author Y. U. on request.

## Conflicts of interest

There are no conflicts to declare.

## Supplementary Material

CB-006-D4CB00243A-s001
