# Supplementary material for: Development of a near-infrared fluorescent probe for the selective detection of severe hypoxia
Source: RSC Chem Biol. 2025 Feb 4;6(3):449–56. doi: 10.1039/d4cb00243a (PMC11791654; doi:10.1039/d4cb00243a)
Supplement: CB-006-D4CB00243A-s001 [file CB-006-D4CB00243A-s001.pdf]

Supplementary Information (SI) for RSC Chemical Biology.
This journal is © The Royal Society of Chemistry 2025

Supporting Information (SI)

Development of near-infrared fluorescence probe for the selective detection of severe hypoxia

Takafumi Kasai, [a] Kyohhei Fujita, [b] Toru Komatsu, [a] Tasuku Ueno, [a] Ryosuke Kojima, [b] Kenjiro Hanaoka,[c] and Yasuteru Urano. [a, b, d]

[a] Graduate School of Pharmaceutical Sciences and [b] Graduate School of Medicine, The University of Tokyo, 7-3-1 Hongo, Bunkyo-ku, Tokyo 113-0033, Japan.

[c] Graduate School of Pharmaceutical Sciences, Keio University, 1-5-30 Shibakoen, Minato-ku, Tokyo 105-8512, Japan.

[d] uranokun@m.u-tokyo.ac.jp

Table of Contents

Figure S1. Chemical structures of MASR and 2,6-diMe azoSiR640. · · · · · · · · · · · · · · · · · · · S3

Figure S2. Reductases reduce azo compounds under hypoxia. · S3

Figure S3. Live cell imaging of hypoxia with T-azoJSiR640 and Tc-azoJSiR640. · · · · · · · · · · · · S3

Figure S4. pH profiles of absorbance and fluorescence spectra of Tc-JSiR640. · · · · · · · · · · · · · S4

Figure S5. Absorbance spectra of Tc-JSiR640 and T-JSiR640 in sodium phosphate buffer (pH 5.0) and CH_2Cl_2. · S4

Figure S6. pH profiles of absorbance and fluoresence spectra of T-JSiR640. · · · · · · · · · · · · · · · S5

Figure S7. Examples of ROIs in live cell fluorescence imaging of A549 cells incubated with T-azoJSiR640. ·

S5

Figure S8. Examples of ROIs in live cell fluorescence imaging of A549 cells incubated with 2,6-diMe azoSiR640. · S6

Figure S9. LC-MS/MS analysis. · S6

Organic synthesis and characterization of compounds. · S7-S11

NMR spectra of compounds. · S12-19

References. · S20

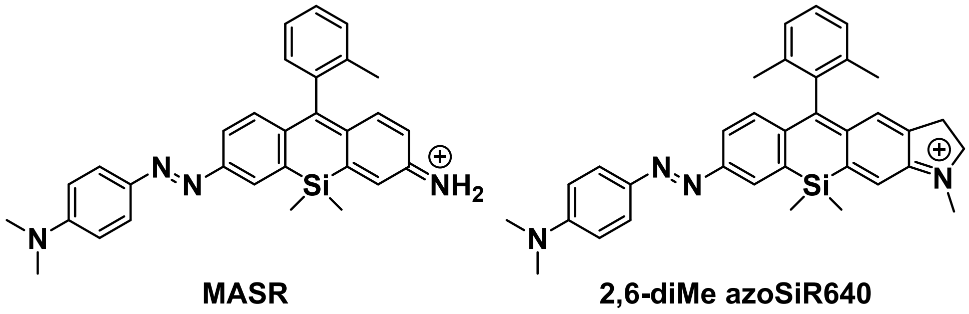

Figure S1. Chemical structures of MASR [1] and 2,6-diMe azoSiR640 [2].

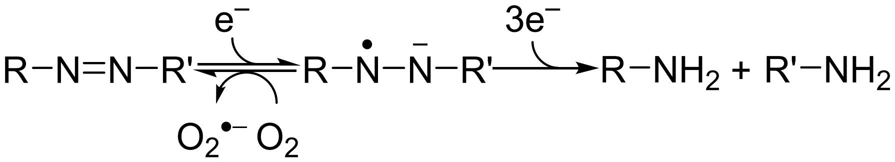

Figure S2. Reductases reduce azo compounds under hypoxia.[1]

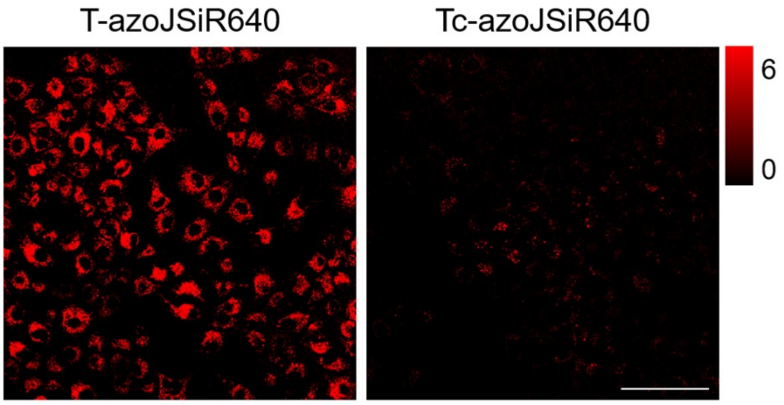

Figure S3. Live cell imaging of hypoxia with T-azoJSiR640 and Tc-azoJSiR640. Fluorescence images of A549 cells incubated with T-azoJSiR640 or Tc-azoJSiR640 at an oxygen concentration under 0.3%. These fluorescence images were captured 6 h after administration of the probe. Imaging was performed in phenol red (-) DMEM containing 10% FBS, 1% PS and 1% DMSO as a cosolvent. The excitation and emission wavelengths were 630 nm/650-750 nm. [T-azoJSiR640] = 1 μM. [Tc-azoJSiR640] = 1 μM. Scale bars = 100 μm.

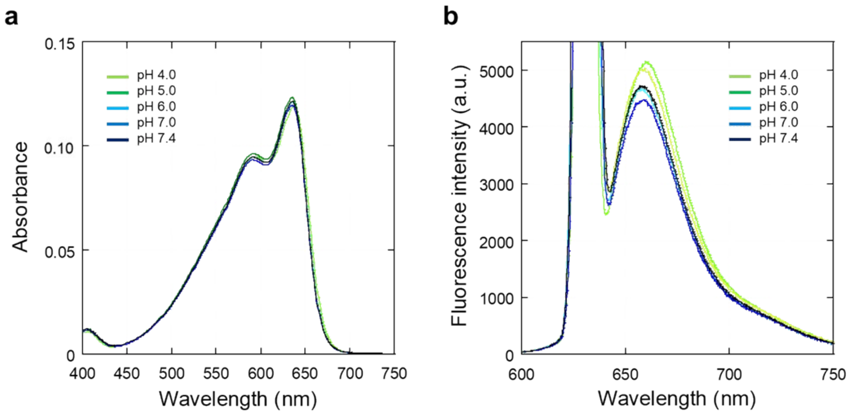

Figure S4. pH profiles of absorbance and fluorescence spectra of Tc-JSiR640. (a) pH dependency of absorbance spectra. [Tc-JSiR640] = 1.0 μM. **(b)** pH dependency of fluorescence spectra. [Tc-JSiR640] = 0.3 μM. Spectra were measured in 100 mM sodium phosphate buffer at pH 4.0-7.4 including 0.1% DMSO. The excitation wavelength was 630 nm. These results show that Tc-JSiR640 can exist in the fluorescent open form at physiological and lysosomal acidic pH.

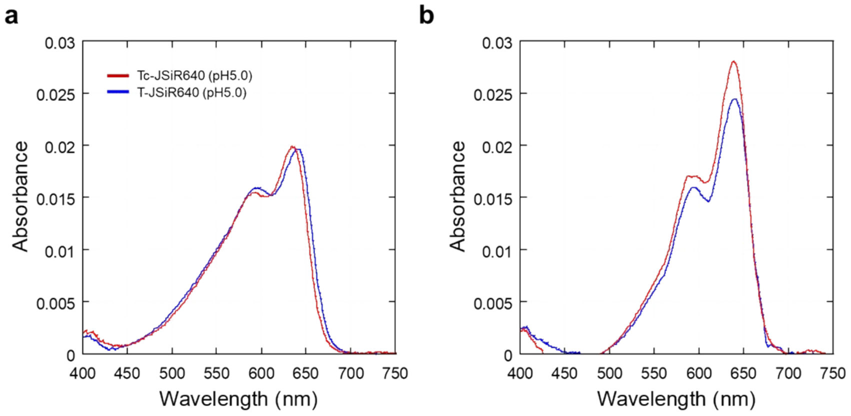

Figure S5. Absorbance spectra of Tc-JSiR640 and T-JSiR640 in sodium phosphate buffer (pH 5.0) and CH_2Cl_2. (a) Spectra were measured in 100 mM sodium phosphate buffer at pH 5.0 including 0.1% DMSO. **(b)** Spectra were measured in CH_2Cl_2. Tc-JSiR640 and T-JSiR640 showed absorbance under both measurement conditions. These results show that Tc-JSiR640 can exist the fluorescent open form in CH_2Cl_2 including 0.1% DMSO, whose polarity is similar to that of internal cell membranes. [Tc-JSiR640] = 0.2 μM. [T-JSiR640] = 0.2 μM.

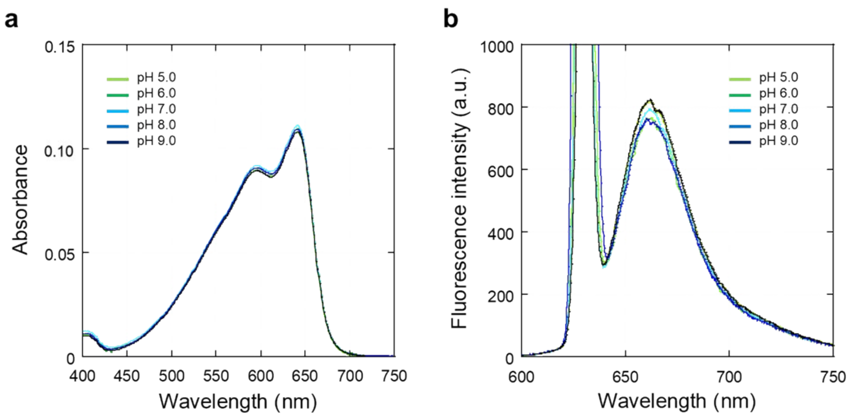

Figure S6. pH profiles of absorbance and fluorescence spectra of T-JSiR640. (a) pH dependency of absorbance spectra. [T-JSiR640] = 1.0 μM. **(b)** pH dependency of fluorescence spectra. [T-JSiR640] = 0.3 μM. Spectra were measured in 100 mM sodium phosphate buffer at pH 5.0-9.0 including 0.1% DMSO. The excitation wavelength was 630 nm.

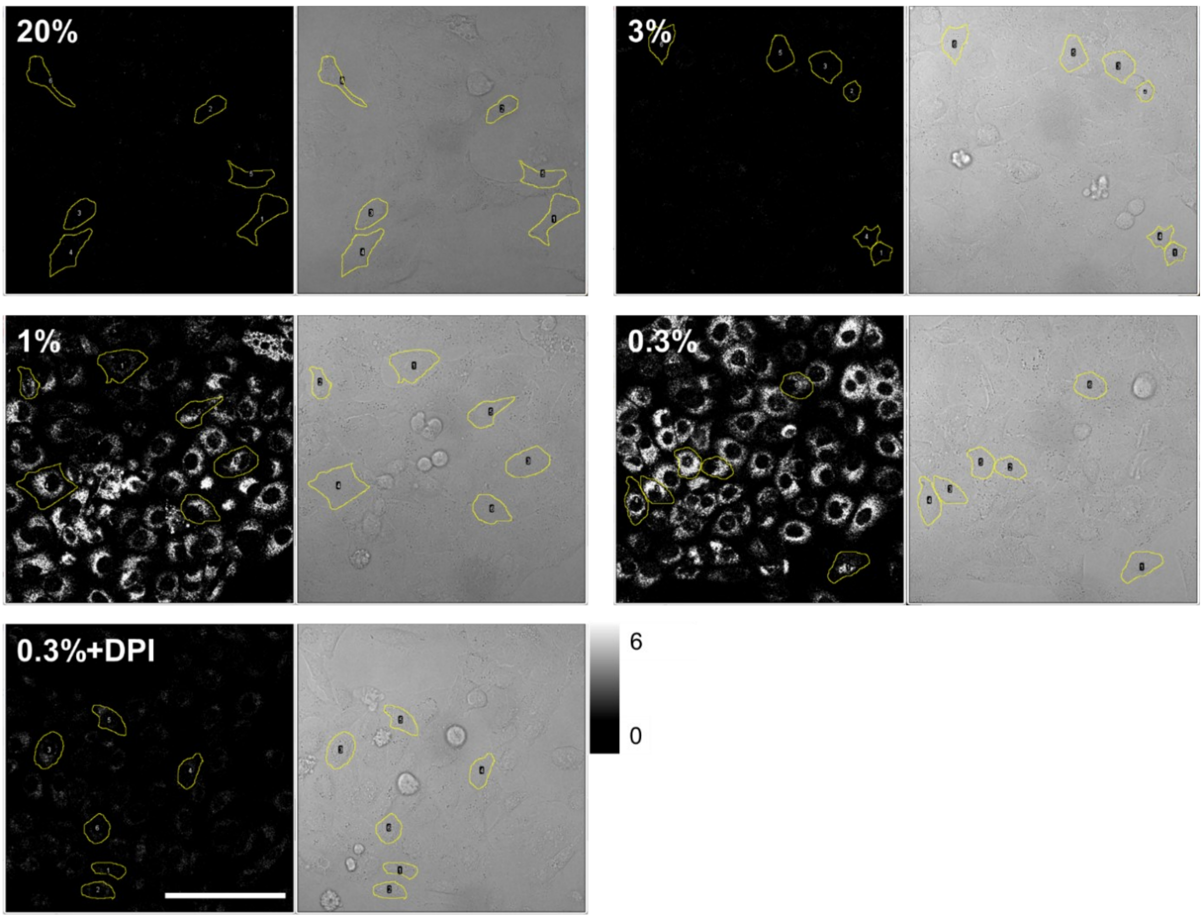

Figure S7. Examples of ROIs in live cell fluorescence imaging of A549 cells incubated with T-azoJSiR640. Four images from different wells were captured under each measurement condition, and six cells per image were randomly selected. Fluorescence intensity of these cells was quantified by drawing regions of interest (ROI) using Image-J

software.

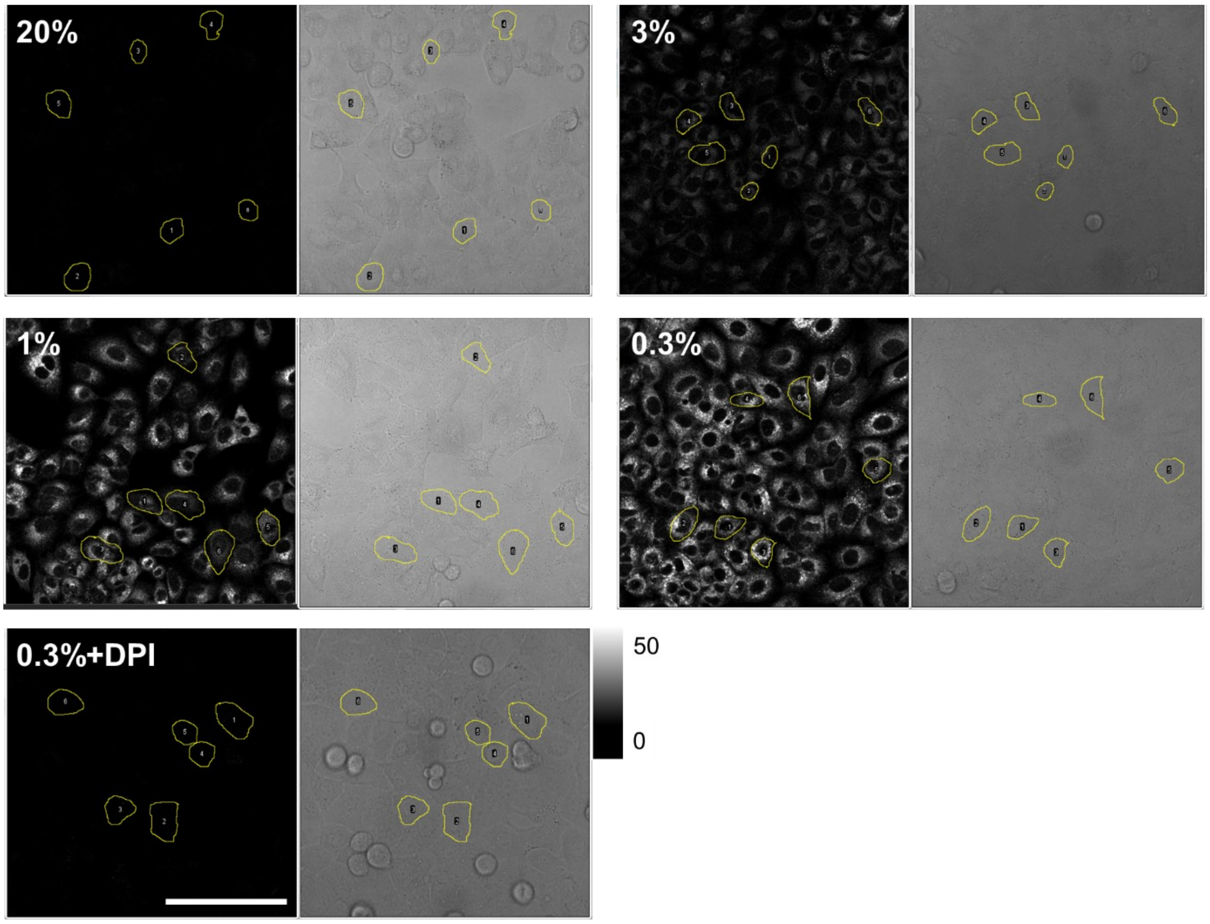

Figure S8. Examples of ROIs in live cell fluorescence imaging of A549 cells incubated with 2,6-diMe azoSiR640. Four images from different wells were captured under each measurement condition, and six cells per image were randomly selected. Fluorescence intensity of these cells was quantified by drawing regions of interest (ROI) using Image-J software.

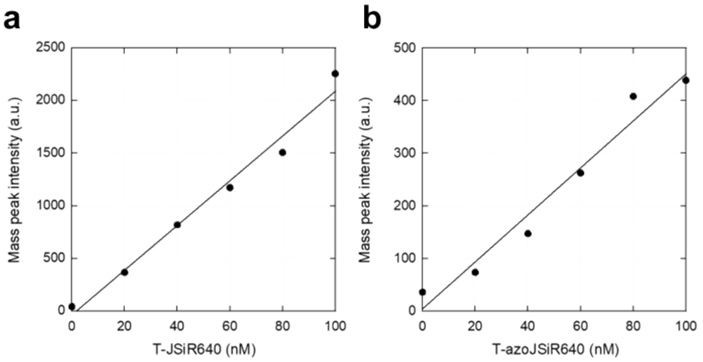

Figure S9. LC-MS/MS analysis. (a) (b) Calibration curves for fluorometric assay of T-JSiR640 and T-azoJSiR640.

Scheme S1. Synthesis of compound 2.

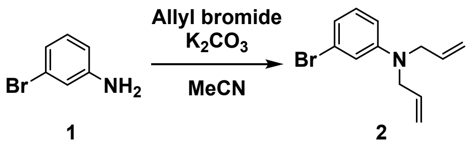

Procedure for the synthesis of compound 2. To a solution of 3-bromoaniline (**1**) (7.9 g, 45.9 mmol) in anhydrous MeCN (40 mL) were added allyl bromide (21.3g, 176 mmol) and K_2CO_3 (19.0 g, 137 mmol). The reaction mixture was stirred for 18 hr at 70°C under an Ar atmosphere. After cooling to room temperature, the mixture was diluted with water, and extracted with AcOEt. The combined organic layer was washed with sat.aq. $NaHCO_3$ and brine, dried over Na_2SO_4, and concentrated-under reduced pressure. The residue was purified by flash column chromatography (silica gel, *n*-hexane/AcOEt =100/0 to 85/15) to afford the desired product **2** (11.4 g, 45.2 mmol) as a yellow oil in 98% yield. The product was identified by means of ^{1}H NMR, ^{13}C NMR and ESI-HRMS.

^{1}H NMR (400 MHz, $CDCl_3$) δ 7.02 (t, *J* = 8.2 Hz, 1H), 6.81-6.77 (m, 2H), 6.58 (dd, *J* = 8.7, 2.7 Hz, 1H), 5.86-5.79 (m, 2H), 5.18-5.14 (m, 4H), 3.91-3.88 (m, 4H).

^{13}C NMR (101 MHz, $CDCl_3$) δ 150.1, 150.0, 149.7, 133.3, 130.4, 123.6, 123.5, 123.2, 119.1, 116.4, 115.0, 110.9, 52.8.

ESI-HRMS (ESI$^+$) *m/z* calcd. for $[M+H]^+$, 252.03879; found, 252.03493 (-3.85 mDa).

Scheme S2. Synthesis of compound 3.

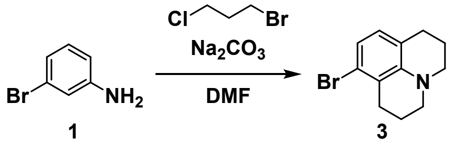

Procedure for the synthesis of compound 3. To a solution of 3-bromoaniline (**1**) (15.9 g, 92.4 mmol) in anhydrous DMF (30 mL) were added 1-bromo-3-chloropropane (60.8 g, 386 mmol) and Na_2CO_3 (49.0g, 462 mmol). The reaction mixture was stirred for 40 hr at 140°C under an Ar atmosphere. After cooling to room temperature, the mixture was filtrered. Sat. aq. $NaHCO_3$ was added to the filtrate and the mixture was extracted with *n*-hexane/AcOEt = 4/1. The combined organic layer was washed with sat. aq. $NaHCO_3$ and brine, dried over Na_2SO_4, and concentrated-under reduced pressure. The residue was purified by flash column chromatography (silica gel, *n*-hexane/AcOEt =100/0 to 85/15) to afford the desired product **3** (4.47 g, 17.7 mmol) as a brown oil in 19% yield. The product was identified by means of ^{1}H NMR, ^{13}C NMR and ESI-HRMS.

^{1}H NMR (400 MHz, $CDCl_3$) δ 6.75 (d, *J* = 7.8 Hz, 1H), 6.64 (d, *J* = 8.2 Hz, 1H), 3.13-3.07 (m, 4H), 2.76 (t, *J* = 6.9 Hz, 2H), 2.69 (t, *J* = 6.9, 2H), 1.98-1.94 (m, 4H).

^{13}C NMR (101 MHz, $CDCl_3$) δ 144.7, 127.9, 123.0, 120.8, 120.7, 119.5, 50.1, 49.7, 28.6, 27.7, 22.0, 21.9.

ESI-HRMS (ESI$^+$) *m/z* calcd. for $[M+H]^+$, 252.03879; found, 252.03485 (-3.93 mDa).

Scheme S3. Synthesis of compound 4.

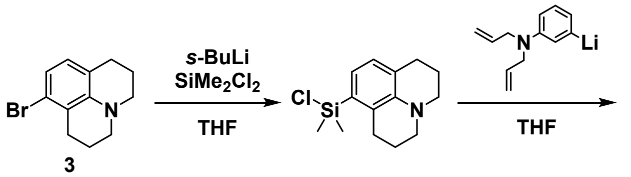

Procedure for the synthesis of compound 4. To a flame-dried flask flushed with Ar atomosphere, **3** (4.47 g, 17.7 mmol) and anhydrous THF (20 mL) were added. The solution was cooled to -78°C and *s*-BuLi (17.7 mL, 1.3 M in cyclohexane-hexane, 23.0 mmol) was added dropwise. The mixture was stirred for 4 hr at -78°C, and a solution of $SiMe_2Cl_2$ (1.9 mL, 19.5 mmol) in anhydrous THF (5 mL) was added. The mixture was warmed to room temperature and evaporated to dryness (**stock 1**). To a flame-dried-flask flushed with Ar atomosphere, **2** (4.55 g, 18.0 mmol) and anhydrous THF (20 mL) were added. The solution was cooled to -78°C, and *s*-BuLi (17.7 mL, 1.3 M in cyclohexane-hexane, 23.0 mmol) was added dropwise. The mixture was stirred for 4 hr at -78°C, and a solution of **stock 1** in anhydrous THF (20 mL) was added dropwise. Stirring was continued at room temperature for 30 min and then the reaction was quenched by the addition of sat. aq. $Na_2S_2O_3$. The mixture was extracted with AcOEt, washed with brine, dried over Na_2SO_4 and concentrated-under reduced pressure. The residue was purified by flash column chromatography (silica gel, *n*-hexane/CH_2Cl_2 = 100/0 to 85/15) to afford the desired product **4** (5.36 g, 13.3 mmol) as a brown oil in 75% yield. The product was identified by means of ^{1}H NMR and ESI-HRMS.

^{1}H NMR (400 MHz, $CDCl_3$) δ 7.16 (m, 1H), 6.86 (d, *J* = 2.7 Hz, 1H), 6.80 (m, 2H), 6.70 (s, 1H), 6.68 (s, 1H), 5.86-5.79 (m, 2H), 5.15-5.10 (m, 4H), 3.89 (m, 4H), 3.13 (t, *J* = 5.5 Hz, 2H), 3.08 (t, *J* = 5.5 Hz, 2H), 2.76 (t, *J* = 6.4 Hz, 2H), 2.70 (t, *J* = 6.4 Hz, 2H), 1.95 (m, 2H), 1.85 (m, 2H), 0.50 (s, 6H).

ESI-HRMS (ESI^+) *m/z* calcd. for $[M+H]^+$,403.25695; found, 403.25558(-1.37 mDa).

Scheme S4. Synthesis of compound 5.

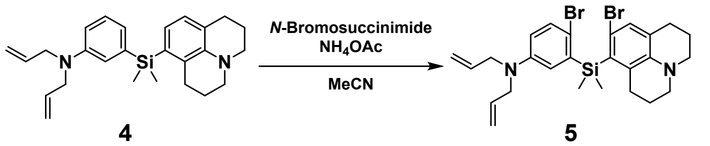

Procedure for the synthesis of compound 5. To a solution of **4** (1.68 g, 4.17 mmol) in anhydrous MeCN (30 mL) was added NH_4OAc (64 mg, 0.83 mmol). The reaction mixture was stirred for 15 min at 0°C under an Ar atmosphere, and *N*-bromosuccinimide (1.48 g, 8.34 mmol) was added. After 3 hr, the reaction mixture was quenched by the addition of sat. aq. $NaHCO_3$ and the mixture was evaporated to remove MeCN. The residue was extracted with AcOEt, washed with brine, dried over Na_2SO_4, and concentrated-under reduced pressure. The residue was purified by flash column chromatography (silica gel, *n*-hexane/AcOEt = 100/0 to 80/20) to afford the desired product **5** (1.58 g, 2.81 mmol) as a brown solid in 67% yield. The product was identified by means of ^{1}H NMR and ^{13}C NMR.

^{1}H NMR (400 MHz, $CDCl_3$) δ 7.22 (m, 1H, partially overlapped with the $CDCl_3$ peak), 6.97 (s, 1H), 6.81 (d, *J* = 3.2 Hz, 1H), 6.51 (dd, *J* = 9.2, 2.7 Hz, 1H), 5.81-5.74 (m, 2H), 5.12-5.05 (m, 4H), 3.84 (m, 4H), 3.11 (t, *J* = 5.6 Hz, 2H),

3.04 (t, $J = 6.0$ Hz, 2H), 2.70-2.66 (m, 4H), 1.91 (m, 2H), 1.77 (m, 2H), 0.73 (s, 6H).

^{13}C NMR (101 MHz, $CDCl_3$) δ 147.0, 142.4, 141.9, 133.8, 132.8, 132.0, 130.6, 125.3, 120.3, 116.6, 116.3, 115.7, 114.7, 53.3, 50.6, 49.6, 31.1, 29.9, 27.9, 22.4, 21.8, 3.74, 0.10.

Scheme S5. Synthesis of compound 6.

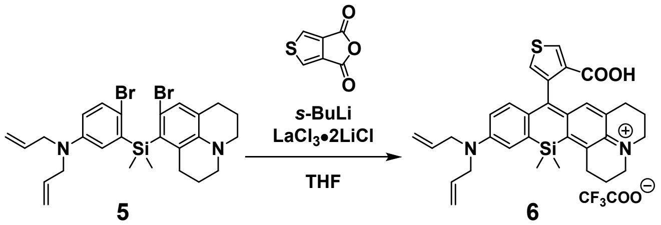

5 6

Procedure for the synthesis of compound 6. This synthesis was conducted based on the reported procedure with modifications.[3] A solution of **5** (420 mg, 0.75 mmol) in anhydrous THF (30 mL) was stirred for 1 hr at -78°C under an Ar atmosphere. To this solution, *s*-BuLi (2.9 mL, 1.3 M in cyclohexane-hexane, 3.77 mmol) was added dropwise. After 15 min, a solution of $LaCl_3$ · 2LiCl (2.8 mL, 0.6 M in THF, 1.68 mmol) was added dropwise, and the reaction mixture was stirred for 2 hr at -78°C. A solution of 3,4-thiophenedicarboxylic anhydride (139 mg, 0.90 mmol) in anhydride THF (5 mL) was added dropwise. The reaction mixture was stirred for 3 hr at -78°C and allowed to warm to room temperature over 2 hr. To this solution was added sat. aq. $NaHCO_3$. The mixture was extracted with AcOEt, washed with brine, dried over Na_2SO_4, and concentrated-under reduced pressure. The residue was purified by HPLC (gradient, eluent A/B = 70/30 to 0/100, 30 min, 5 mL/min) to afford the desired product **6** (229 mg, 0.35 mmol) as a blue powder in 47% yield. The product was identified by means of ^{1}H NMR, ^{13}C NMR and ESI-HRMS.

^{1}H NMR (400 MHz, CD_3OD) δ 8.46 (d, $J = 3.2$ Hz, 1H), 7.35 (d, $J = 3.2$ Hz, 1H), 7.09 (d, $J = 3.2$ Hz, 1H), 6.92 (d, $J = 9.6$ Hz, 1H), 6.85 (s, 1H), 6.69 (dd, $J = 11.0$, 2.7 Hz 1H), 5.90-5.86 (m, 2H), 5.25-5.14 (m, 4H), 4.18 (m, 4H), 3.61 (m, 4H), 3.00 (m, 2H), 2.52 (m, 2H), 2.07 (m, 2H), 1.91 (m, 2H), 0.584 (s, 3H), 0.580 (s, 3H).

^{13}C NMR (101 MHz, CD_3OD) δ 163.7, 163.1, 152.4, 151.6, 145.6, 142.3, 140.3, 139.4, 138.4, 134.9, 134.7, 133.2, 131.9, 128.7, 128.5, 126.4, 124.3, 119.3, 116.1, 113.5, 52.9, 52.0, 51.3, 28.6, 27.1, 20.6, 20.2, -1.39, -2.22, -2.32.

ESI-HRMS (ESI$^+$) *m/z* calcd. for $[M+H]^+$, 539.21885; found, 539.21854(-0.31 mDa).

Scheme S6. Synthesis of compound 7.

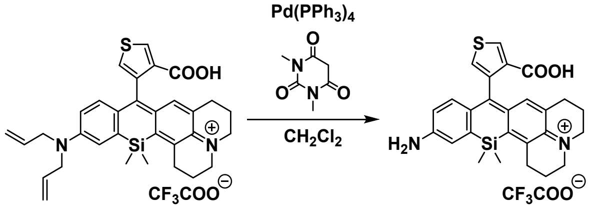

6 7

Procedure for the synthesis of compound 7. To a solution of **6** (75 mg, 0.11 mmol) in deoxidized CH_2Cl_2 (10 mL) was added 1,3-dimethylbarbituric acid (90 mg, 0.58 mmol) and $Pd(PPh_3)_4$ (27 mg, 0.023 mmol) under an Ar atmosphere. The reaction mixture was stirred for 5 hr at 35°C. After the reaction, the mixture was concentrated in vacuo. The residue was purified by HPLC (gradient, eluent A/B = 90/10 to 0/100, 30 min, 5.0 mL/min) to afford the

desired product **7** (48 mg, 0.084 mmol) as a blue powder in 73% yield. The product was identified by means of ^{1}H NMR, ^{13}C NMR and ESI-HRMS.

^{1}H NMR (400 MHz, CD_3OD) δ 8.47 (d, J = 3.6 Hz, 1H), 7.36 (d, J = 3.2 Hz, 1H), 7.07 (d, J = 2.3 Hz, 1H), 6.89 (d, J = 9.2 Hz, 1H), 6.84 (s, 1H), 6.53 (dd, J = 9.2, 2.4 Hz, 1H), 3.60 (m, 4H), 3.02 (t, J = 5.2 Hz, 2H), 2.53 (t, J = 6.0 Hz, 2H), 2.09 (m, 2H), 1.92 (m, 2H), 0.602 (s, 3H), 0.598 (s, 3H).

^{13}C NMR (101 MHz, CD_3OD) δ 164.3, 163.1, 155.4, 151.1, 147.1, 141.8, 140.4, 139.6, 139.3, 134.83, 134.78, 132.7, 128.5, 128.2, 126.4, 123.9, 121.1, 114.9, 51.8, 51.1, 28.6, 27.1, 20.6, 20.3, -2.31, -2.37.

ESI-HRMS (ESI^+) *m/z* calcd. for $[M+H]^+$,459.15625; found, 459.15412(-2.13 mDa).

Scheme S7. Synthesis of compound 8 (T-JSiR640).

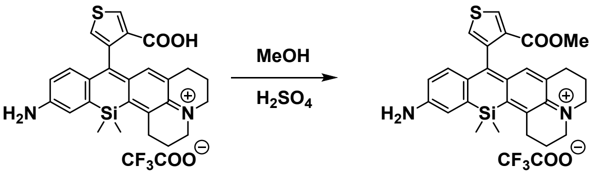

7 **8, T-JSiR640**

Procedure for the synthesis of compound 8 (T-JSiR640). To a solution of **7** (19.7 mg, 0.034 mmol) in anhydrous MeOH (20 mL) was added one drop of H_2SO_4. The reaction mixture was stirred for 23 hr at 70°C under an Ar atmosphere and cooled to 0°C, then water was added and the mixture was evaporated. The residue was purified by HPLC (gradient, eluent A/B = 95/5 to 0/100, 30 min, 5mL/min) to afford the desired product **8** (9.2 mg, 0.016 mmol) as a blue powder in 46% yield. The product was identified by means of ^{1}H NMR, ^{13}C NMR and ESI-HRMS.

^{1}H NMR (400 MHz, CD_3OD) δ 8.51 (d, J = 3.2 Hz, 1H), 7.40 (d, J = 3.6 Hz, 1H), 7.08 (d, J = 2.0 Hz, 1H), 6.85 (d, J = 9.2 Hz, 1H), 6.81 (s, 1H), 6.52 (dd, J = 9.2, 2.4 Hz 1H), 3.63 (m, 3H, partially overlapped with the 3.59 ppm peak), 3.59 (m, 4H), 3.03 (t, J = 5.6 Hz, 2H), 2.52 (t, J = 6.0 Hz, 2H), 2.09 (m, 2H), 1.92 (m, 2H), 0.62 (s, 3H), 0.61 (s, 3H).

^{13}C NMR (101 MHz, CD_3OD) δ 140.5, 140.2, 135.9, 127.5, 125.0, 122.2, 115.9, 52.8, 52.1, 52.0, 29.6, 28.1, 21.6, -0.39, -1.30, -1.36.

ESI-HRMS (ESI^+) *m/z* calcd. for $[M+H]^+$, 473.17190; found, 473.17032(-1.58 mDa).

Scheme S8. Synthesis of compound 9 (T-azoJSiR640).

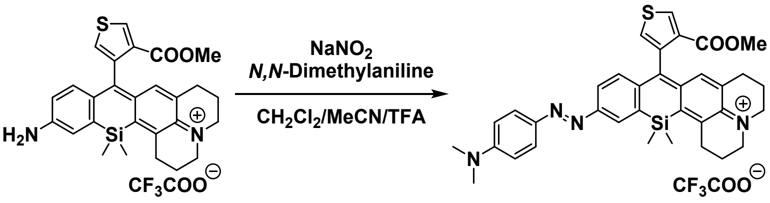

8 **9, T-azoJSiR640**

Procedure for the synthesis of compound 9 (T-azoJSiR640). A solution of **8** (4.7 mg, 8.0×10^{-3} mmol) in CH_2Cl_2/MeCN/TFA (=8/1/0.1 mL) was stirred for 10 min at -5°C under an Ar atmosphere. To this solution, $NaNO_2$ (1.1 mg, 1.6×10^{-2} mmol) was added. The reaction was continued at -5°C for 1 hr and *N,N*-dimethylaniline (1.5 µL, 1.2×10^{-2} mmol) in MeCN (1 mL) was added. After further stirring at -5°C for 3 hr, water was added and the mixture

was evaporated. The residue was purified by HPLC (gradient, eluent C/E = 70/30 to 0/100, 30 min, 7 mL/min) and (gradient, eluent A/D = 70/30 to 0/100, 20 min, 7 mL/min) to afford the desired product **9** (2.0 mg, 2.8×10^{-3} mmol) as a dark blue powder in 35% yield. The product was identified by means of ^{1}H NMR, ESI-HRMS. The reaction was monitored by HPLC.

^{1}H NMR (400 MHz, CD_3OD) δ 8.58 (d, *J* = 3.2 Hz, 1H), 8.20 (d, *J* = 2.3 Hz, 1H), 7.88 (m, 2H), 7.67 (dd, *J* = 8.7, 1.8 Hz, 1H), 7.52 (d, *J* = 3.2 Hz, 1H), 7.07 (d, *J* = 8.7 Hz, 1H), 7.04 (s, 1H), 6.86 (d, *J* = 9.2 Hz, 2H), 3.82 (m, 4H), 3.63 (s, 3H), 3.14 (m, 2H), 2.61 (m, 2H), 2.18 (m, 2H), 1.99 (m, 2H), 1.31 (t, *J* = 7.3 Hz, 6H), 0.75 (s, 3H), 0.73 (s, 3H).

ESI-HRMS (ESI^+) *m/z* calcd. for $[M+H]^+$, 605.24065; found, 605.23955(-1.10 mDa).

Scheme S9. Synthesis of compound 10 (Tc-azoJSiR640).

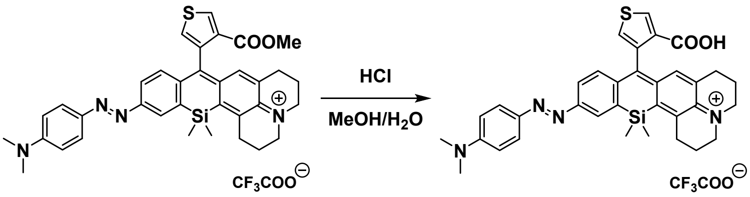

9, T-azoJSiR640 **10, Tc-azoJSiR640**

Procedure for the synthesis of compound 10 (Tc-azoJSiR640). To a solution of **9, T-azoJSiR640** (6.2 mg, 8.6×10^{-3} mmol) in $MeOH/H_2O$ (1/20 mL) was added HCl (1 mL). The reaction mixture was stirred at reflux under an Ar atomosphere for 16.5 hr and evaporated. The residue was purified by HPLC (gradient, eluent C/E = 70/30 to 0/100, 30 min, 7 mL/min) and (gradient, eluent A/D = 70/30 to 0/100, 20 min, 7 mL/min) to afford the desired product **10** (0.7 mg, 9.9×10^{-4} mmol) as a blue-black powder in 12% yield. The product was identified by means of ^{1}H NMR, ESI-HRMS. The reaction was monitored by HPLC.

^{1}H NMR (400 MHz, CD_3OD) δ 8.55 (d, *J* = 3.7 Hz, 1H), 8.18 (d, *J* = 2.3 Hz, 1H), 7.89 (s, 1H), 7.87 (s, 1H), 7.70 (dd, *J* = 8.7, 1.8Hz, 1H), 7.48 (d, *J* = 3.2 Hz, 1H), 7.12 (d, *J* = 9.2 Hz, 1H), 7.07 (s, 1H), 6.87 (s, 1H), 6.84 (s, 1H), 3.80 (m, 4H), 3.14 (m, 2H), 2.62 (m, 2H), 2.18 (m, 2H), 2.00 (m, 2H), 1.31 (t, 6H), 0.73 (s, 3H), 0.71 (s, 3H).

ESI-HRMS (ESI^+) *m/z* calcd. for $[M+H]^+$,591.22500 ; found, 591.22555 (0.55 mDa).

Scheme S10. Synthesis of 2,6-diMe azoSiR640. 2,6-diMe azoSiR640 was synthesized according to the reported procedure.[2]

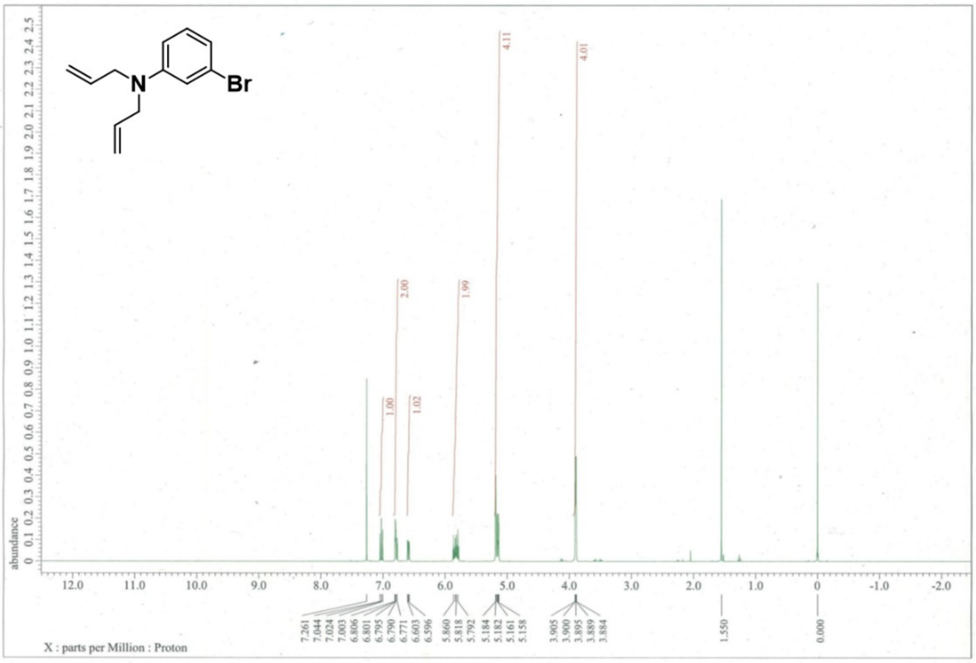

^{1}H NMR spectrum of compound 2.

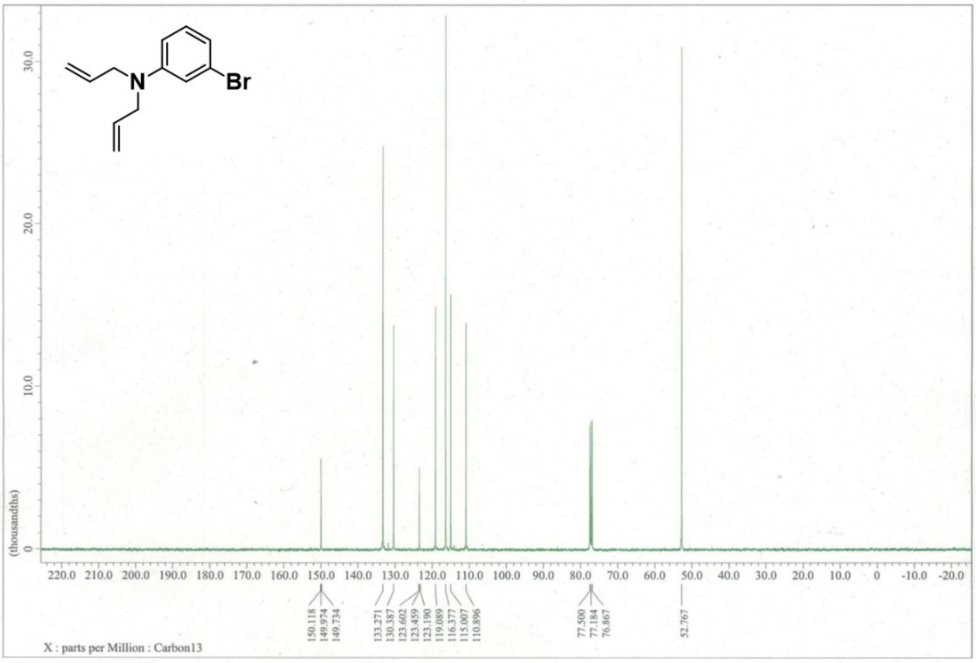

^{13}C NMR spectrum of compound 2.

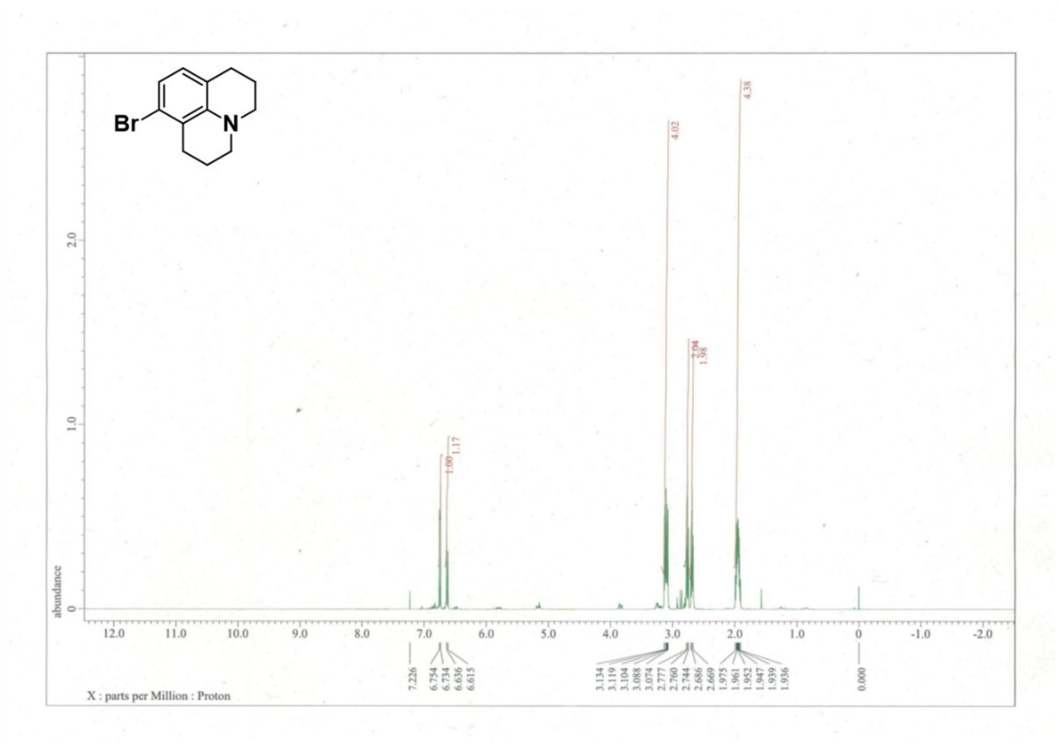

¹H NMR spectrum of compound 3.

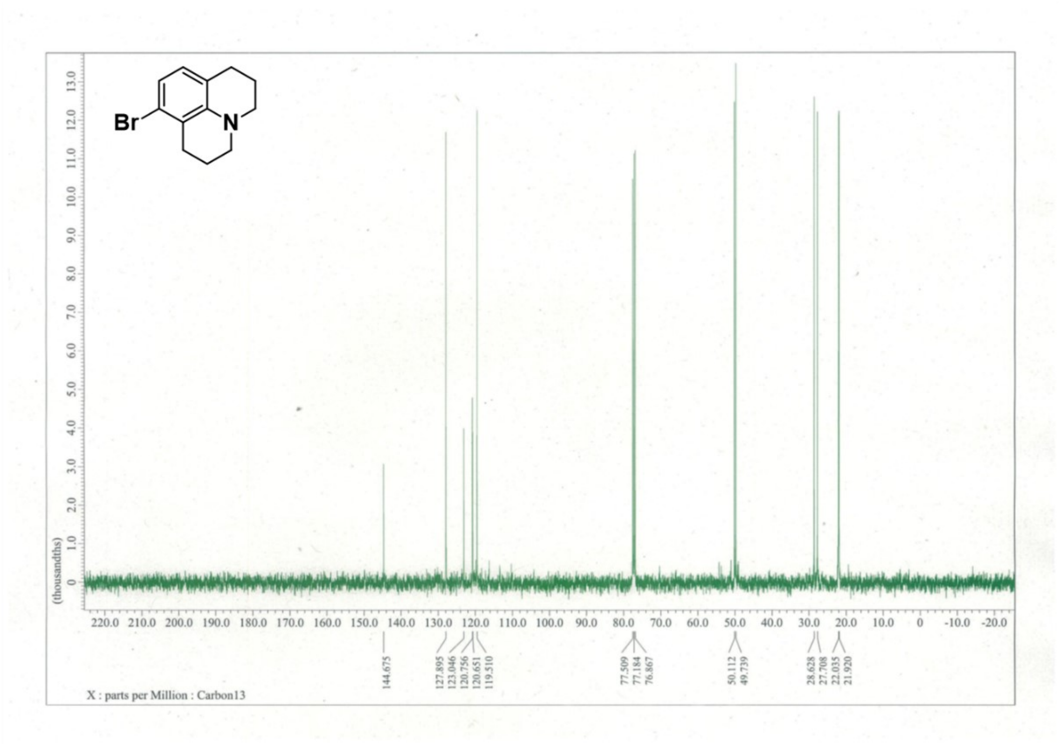

¹³C NMR spectrum of compound 3.

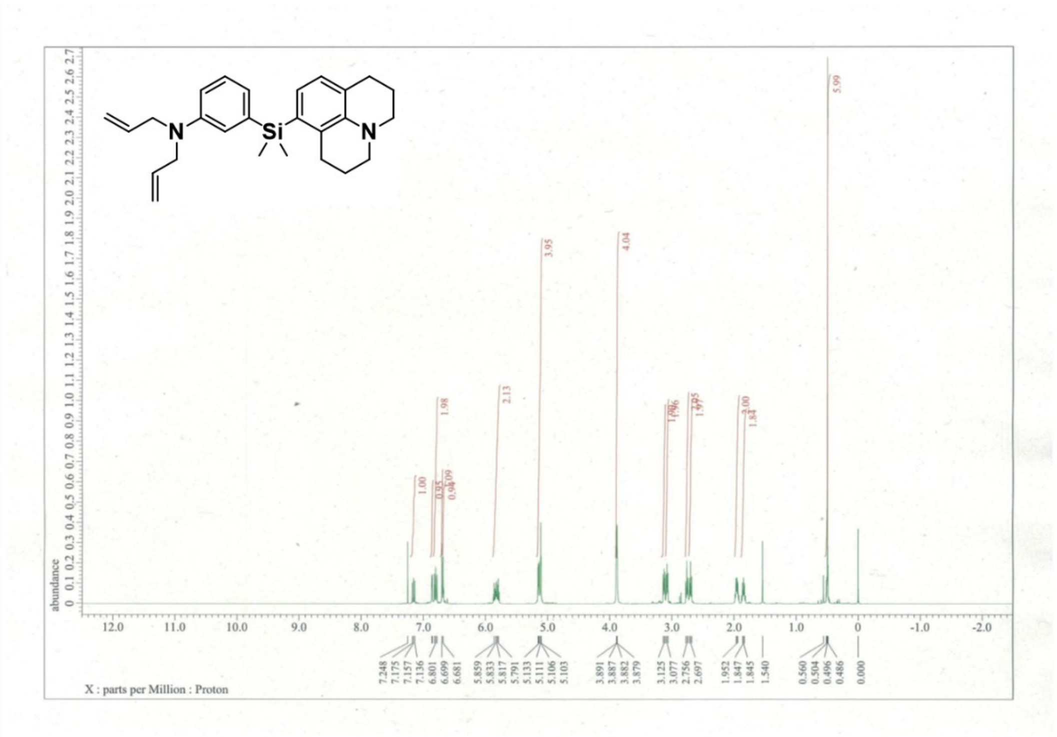

^1H NMR spectrum of compound 4.

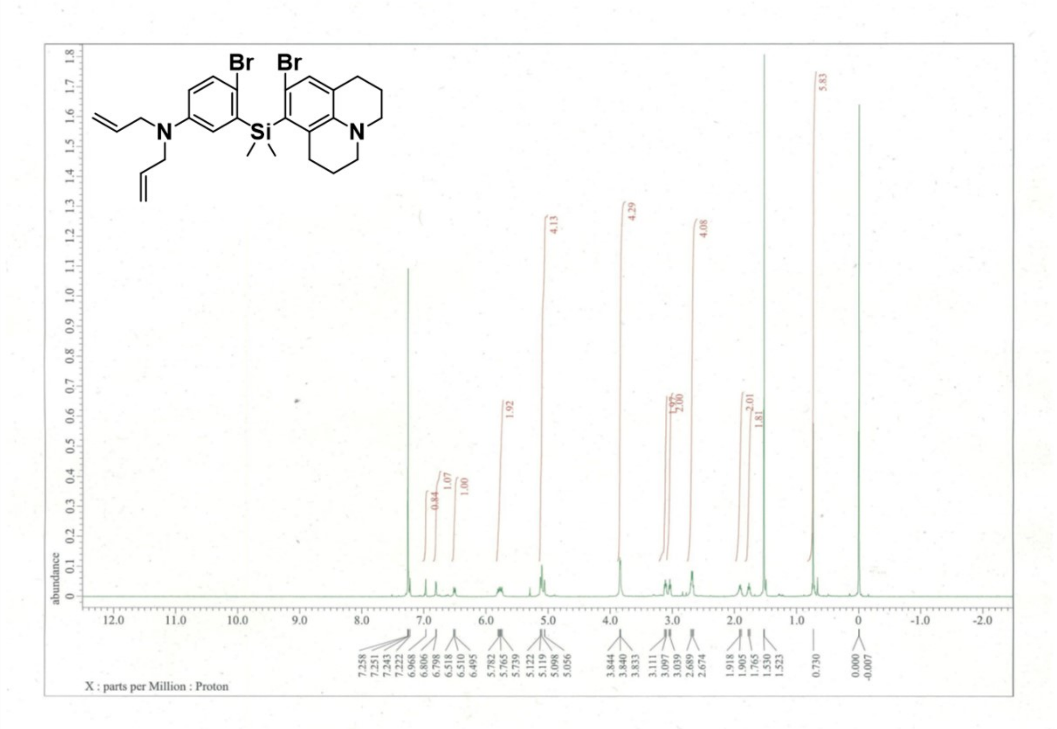

^{1}H NMR spectrum of compound 5.

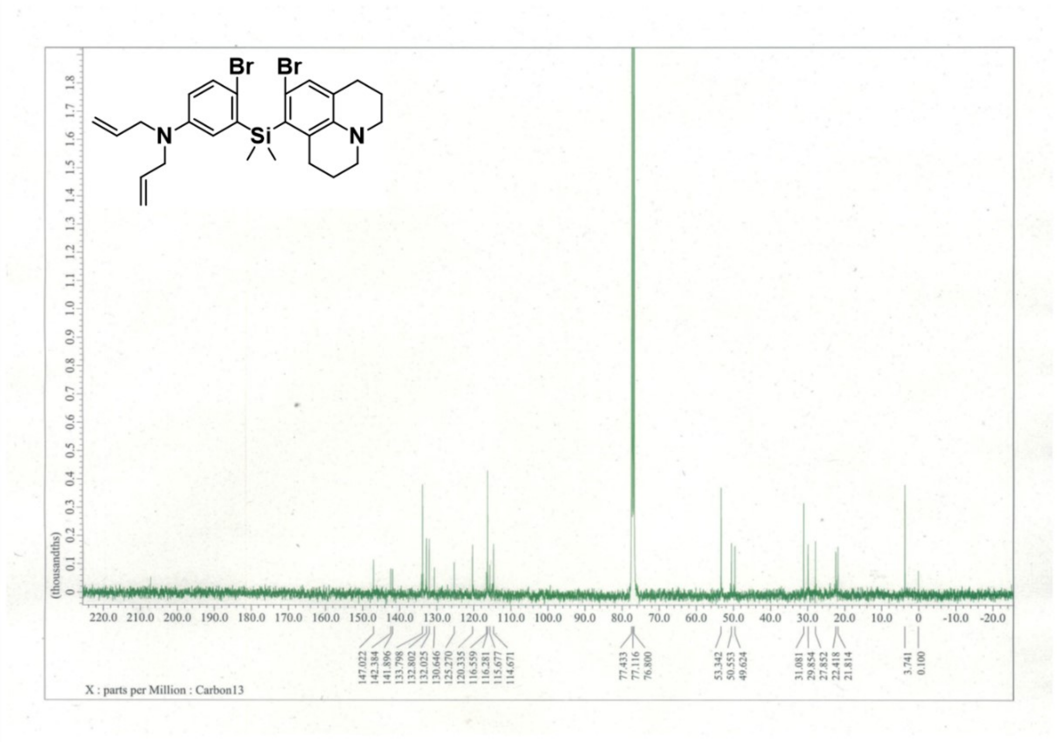

^{13}C NMR spectrum of compound 5.

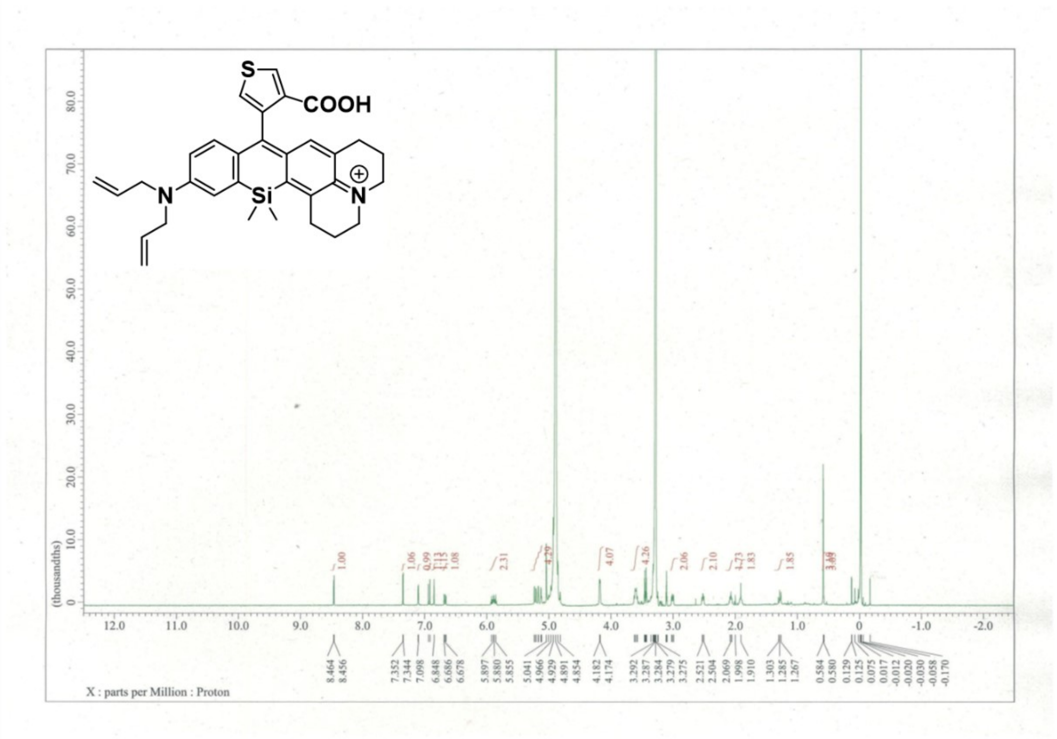

^{1}H NMR spectrum of compound 6.

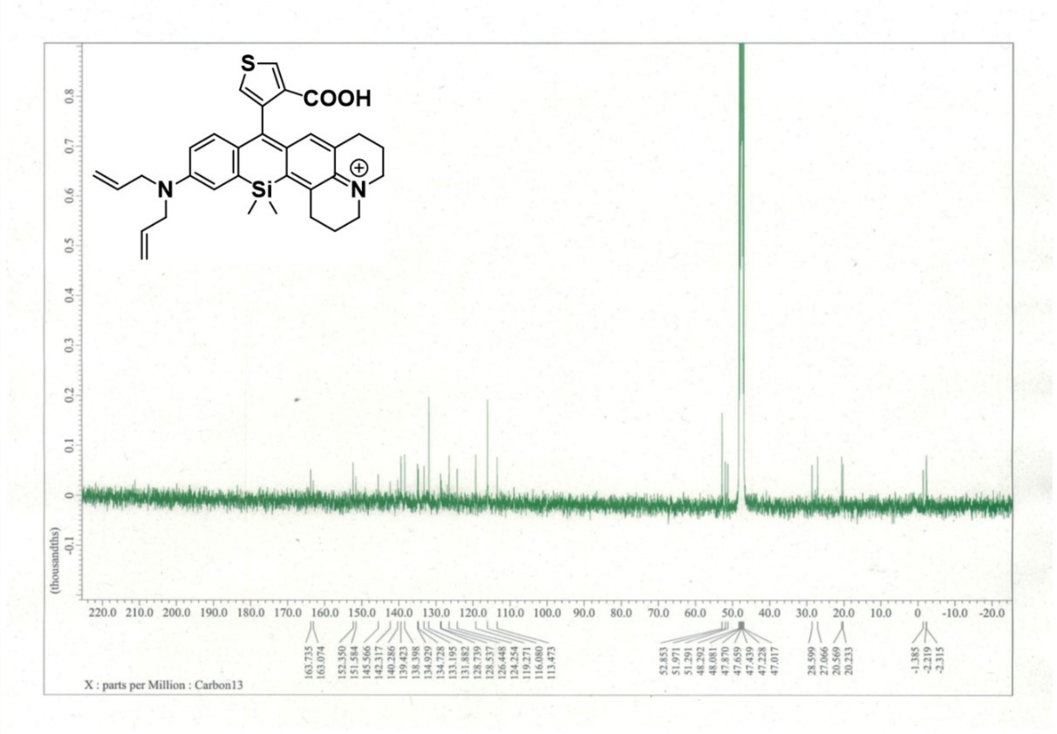

^{13}C NMR spectrum of compound 6.

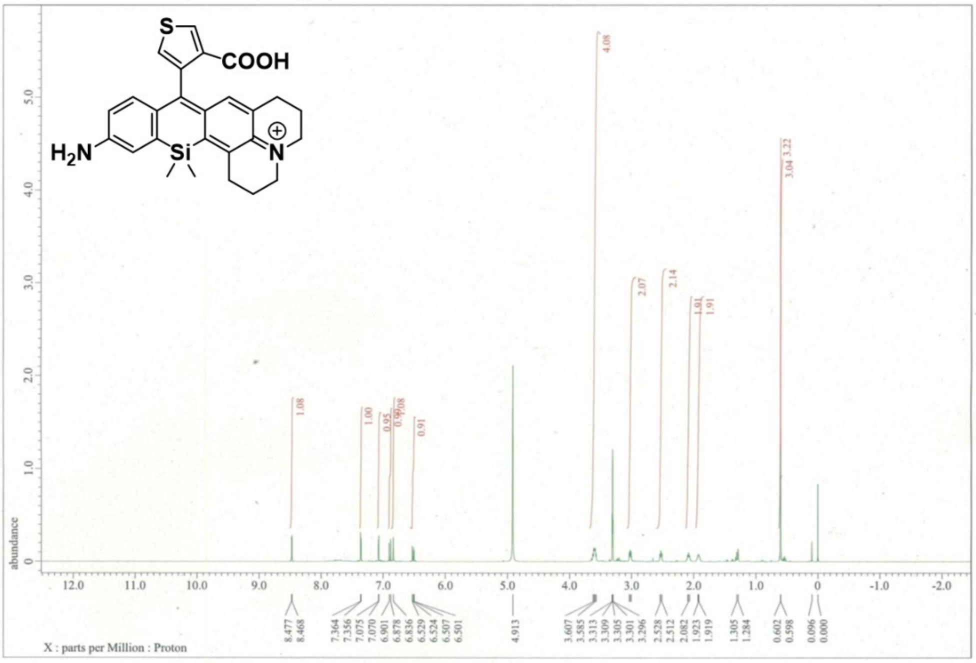

^{1}H NMR spectrum of compound 7.

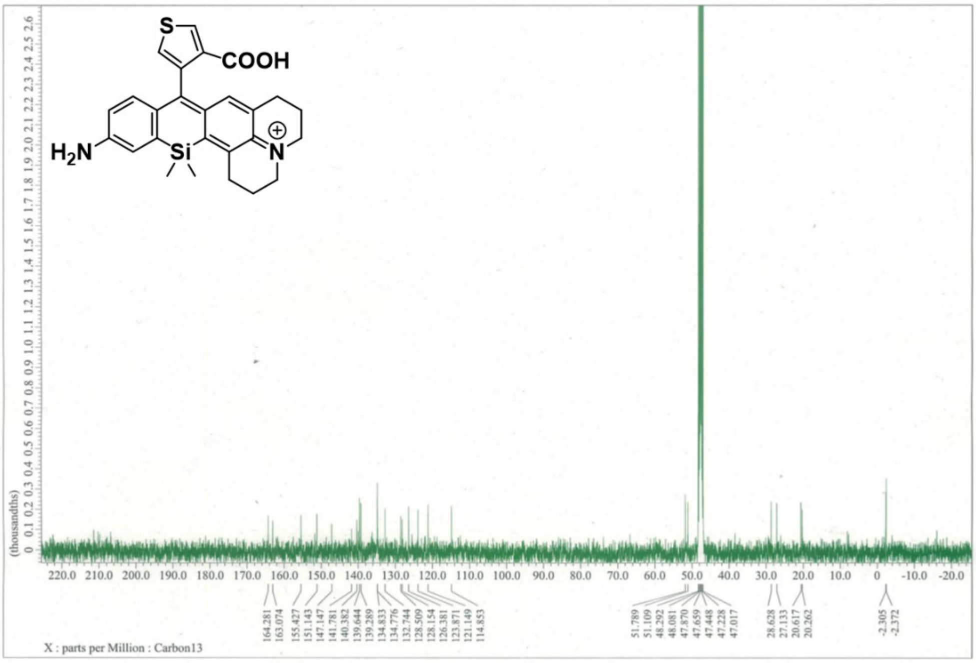

^{13}C NMR spectrum of compound 7.

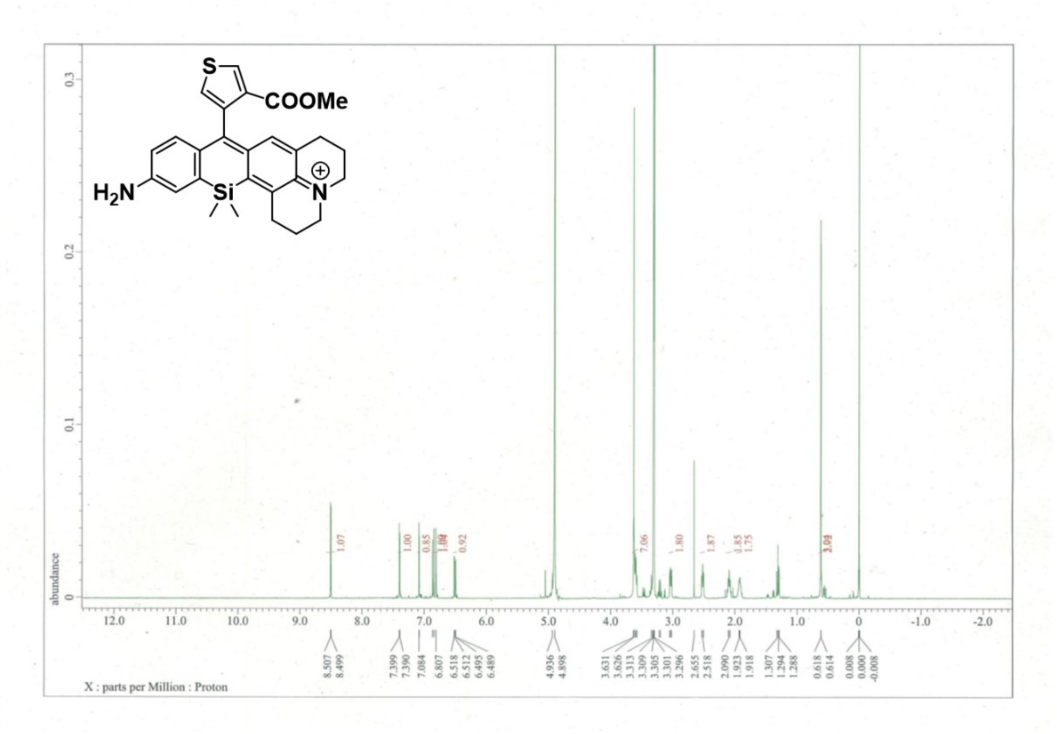

^{1}H NMR spectrum of compound 8.

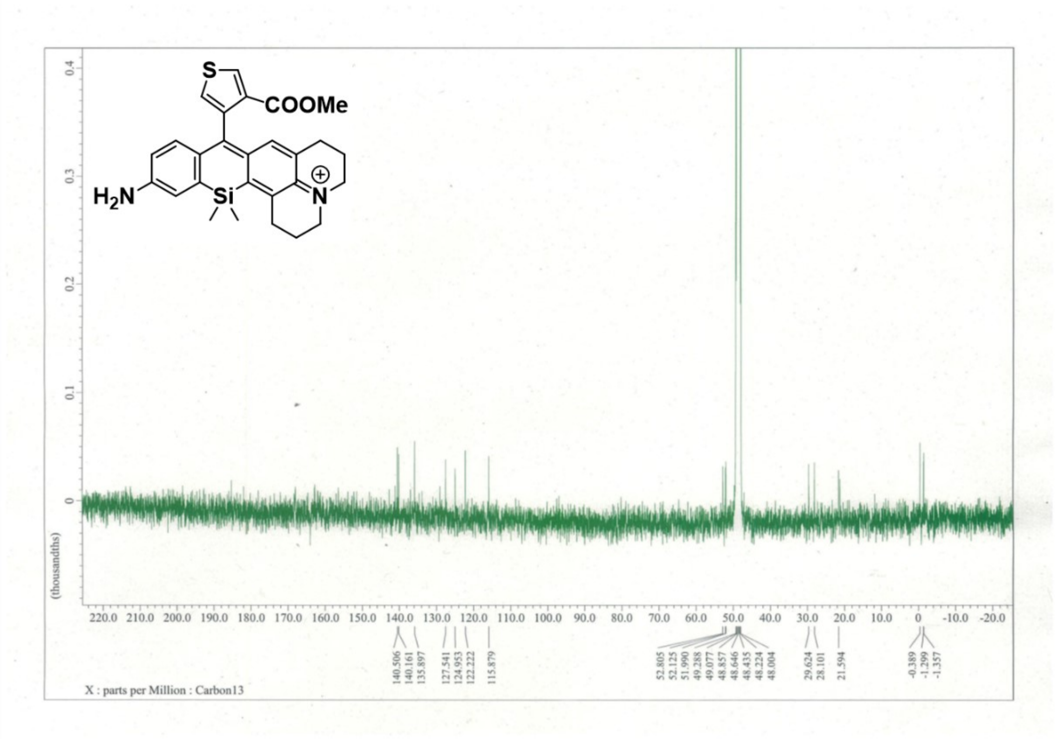

^{13}C NMR spectrum of compound 8.

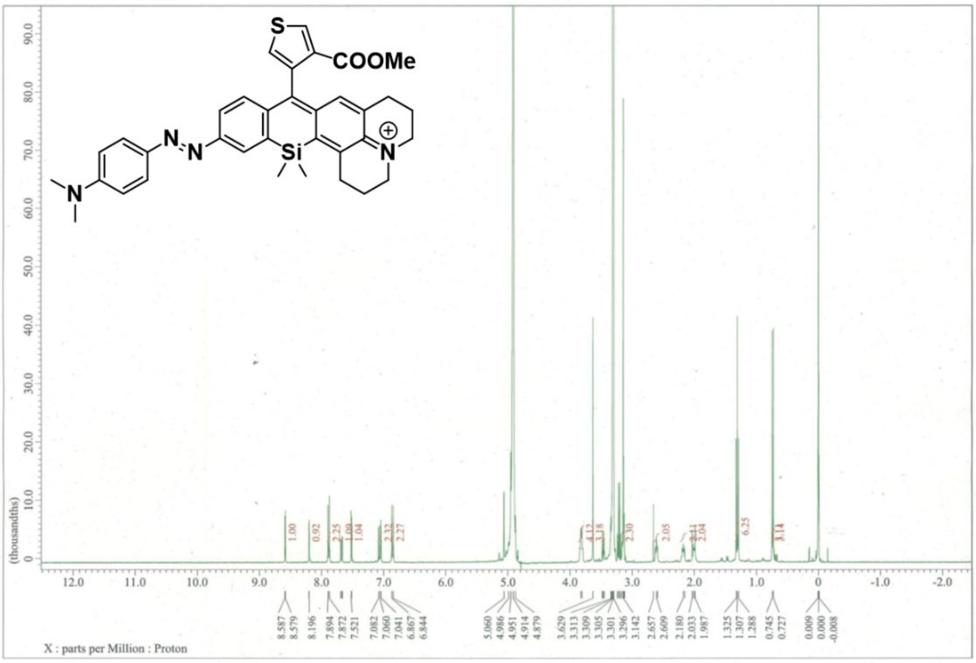

^{1}H NMR spectrum of compound 9.

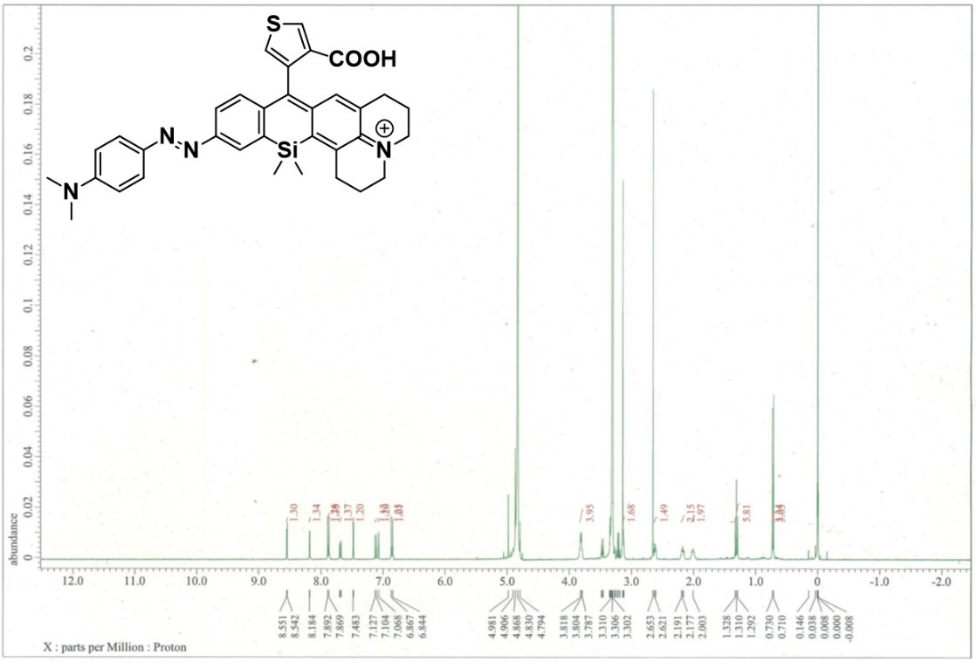

^{1}H NMR spectrum of compound 10.

References

1. Piao, W. Tsuda, S., Tanala, Y., Maeda, S., Liu, F., Takahashi, S., Kushida, Y., Komatsu, T., Ueno, T., Terai, T., Nakazawa, T., Uchiyama, M., Morokuma, K., Nagano, T. & Hanaoka, K. (2013) Development of Azo-Based Fluorescent Probes to Detect Different Levels of Hypoxia. *Angew. Chem. Int. Ed.*, **52**, 13028–13032.
2. Hanaoka, K. Kagami, Y., Piao. W., Myochin, T., Numasawa, K., Kuriki, Y., Ikeno, T., Ueno, T., Komatsu, T., Terai, T., Nagano, T. & Urano, Y. (2018) Synthesis of unsymmetrical Si-rhodamine fluorophores and application to a far-red to near-infrared fluorescence probe for hypoxia. *Chem. Commun.*, **54**, 6939–6942.
3. Butkevich, A. N. (2021) Modular Synthetic Approach to Silicon-Rhodamine Homologues and Analogues via Bis-aryllanthanum Reagents. *Org. Lett.*, **23**, 2604-2609.
